# Strawberry Proteome Responses to Controlled Hot and Cold Stress Partly Mimic Post-harvest Storage Temperature Effects on Fruit Quality

**DOI:** 10.3389/fnut.2021.812666

**Published:** 2022-02-15

**Authors:** Jinhua Lv, Ting Zheng, Zenglu Song, Tariq Pervaiz, Tianyu Dong, Yanyi Zhang, Haifeng Jia, Jinggui Fang

**Affiliations:** ^1^College of Horticulture, Nanjing Agricultural University, Nanjing, China; ^2^Institute of Horticulture, Zhejiang Academy of Agricultural Sciences, Hangzhou, China; ^3^College of Electrical Engineering, Nanjing Vocational University of Industry Technology, Nanjing, China; ^4^Agricultural College, Liaocheng University, Liaocheng, China

**Keywords:** *Fragaria* × *ananassa*, post-harvest fruit quality, storage temperature, proteomic profiling, protein function

## Abstract

To determine the effect of different temperature on strawberry after harvest, physiological indicator analysis and proteomics analysis were conducted on ripened strawberry (“Sweet Charlie”) fruit stored at 4, 23, and 37°C for 10 or 20 days. Results showed that 4°C maintained a better visual quality of strawberry, and the weight loss and firmness remained stable within 3 days. Low temperature negatively affected anthocyanin but positively affected soluble sugars. Though anthocyanin content was higher with increasing temperature, anthocyanin synthesis related proteins were downregulated. Higher indole-acetic acid (IAA) content in seeds and lower abscisic acid (ABA) content were found in berry at 4°C. Antioxidant related proteins were upregulated during storage, showing a significant up-regulation of peroxidase (POD) at 4°C, and ascorbate-glutathione (AsA-GSH) cycle related proteins and heat shock proteins (HSPs) at 37°C. In addition, overexpressed sugar phosphate/phosphate translocator, 1-aminocyclopropane-1-carboxylate oxidase, and aquaporin PIP2-2 had a positive effect in response to low temperature stress for containing higher protopectin content and POD activity.

## Introduction

Strawberry (*Fragaria* × *ananassa* Duch.) is one of the most important globally cultivated fruit crops. Strawberry fruit is widely consumed not only for its flavor and appearance but also for its medicinal value, such as eyesight protection, improved blood circulation (1), and anticarcinogenic activity (2). Unfortunately, strawberries are perishable and susceptible to mechanical injury and physiological deterioration, which cause serious economic loss. Too high or too low storage temperature after harvest will have an important impact on the internal and external quality of strawberry fruit (3). At present, some researchers have made in-depth studies on the mechanism of low-temperature storage of strawberry after harvest (4, 5). The evaluation of strawberry quality for the market is focused on visual and internal characteristics, such as color, hardness, acidity, sweetness, and aroma (6). With the development of economy, more, and more people are concerned about the nutritional quality of strawberries. Strawberries are good sources of natural antioxidants and its antioxidant activity are positively correlated with anthocyanin or total phenol content (7, 8). In addition to its antioxidant activity, anthocyanins are also important to fruit color during the ripening of strawberry fruit, grapes, and cherries (3, 9, 10). Strawberry aroma is one of the most popular fruit flavors, and the flavor strawberries is mainly determined by a complex mixture of esters, aldehydes, alcohols, and sulfur compounds (5, 11). Among the identified aromatic compounds, esters, and furanones are consider the main aroma determinants in fresh strawberry.

Changes of physiological and molecular occurred in plant in response to low or high temperature have been extensively studied (5, 12–14). HSPs are stress proteins with anti-stress effects, which can be expressed in large quantities under stress such as high temperature, drought, peroxidation, and heavy metals (15). Soluble sugars, amino acids, organic acids are important compatible substances, which played crucial role in the resistance of plants to environmental changes (16, 17). The richer the osmotic adjustment substances, the better the adaptability to the environment. Softening of fruit is closely related to the disassembly of primary cell wall and middle lamella structures and the decrease of soluble pectin (18). Studies have shown that the changes in the composition and structure of the cell wall are caused by the coordinated action of hydrolytic enzymes (19).

Temperature regulation is the most important environmental factor for extending the shelf-life of strawberry fruit. Cordenunsi et al. (20) reported that the quality of strawberry fruit was maintained better at 6°C for 6 days than at 16 and 25°C, and low temperature inhibited the accumulation of anthocyanins and vitamin C, but promoted soluble sugars. Higher storage temperatures increase respiration rates and shorten storage life, resulting in fruit quality loss (5, 21, 22). Strawberry fruit stored in different relative humidity (RH) environments at 0.5, 10, and 20°C for 4 days were studied by Shin et al. (22), who found that firmness and soluble solid concentrations decreased at higher storage temperature, while anthocyanin concentrations were increased rapidly at 20°C as the fruit ripened.

Proteomics is a kind of discipline that studies the composition of cell proteins and the various activity characteristics of proteins (23, 24). Compared with genome, proteomics can provide a direct basis for explaining the essence of life phenomena. In proteomics, Mass Spectrometry (MS) technology is the core content of its research, mainly to realize its molecular identification, modification, and the interaction mechanism between various molecules through the correct determination of the mass of the protein molecule (25). At present, researchers are devoted to the study of the biological characteristics of fruits during the pre-harvest and post-harvest storage processes (26). However, there are not many research data on the proteomics of fruit changes (27, 28).

The objective of our study was to investigate the response mechanism of strawberry fruit harvested at the red ripe stage to different temperatures. Although the effect of temperature on the quality of strawberry has been studied by some researchers (5, 9, 22); however, not all of the physiological quality data were assessed at the same time, and limited research has been conducted on protein expression. In addition, previous studies have shown that the response temperature of strawberry heat shock protein is generally 30–37°C (29). Under low temperature storage conditions, strawberry fruits could maintain acceptable quality for up to 7 days, but for high temperature stress, the time is much shorter (5). Therefore, strawberry fruits treated at 4°C, 23°C (simulated room temperature), and 37°C (heat shock temperature) on day 3, 7, 10, and/or 20 were collected for determination.

## Materials and Methods

### Plant Material and Growth Conditions

Octoploid strawberry (*Fragaria* × *ananassa* “Sweet Charlie”) were grown in a greenhouse under standard cultivation conditions (20–25°C, relative humidity of 70–85%, 14 h/10 h light/dark cycles) during spring seasons from 2018 to 2020. According to our previous research, we divide strawberry development into 7 stages [SG (Small green, 7 days after anthesis), LG (Large green, 12 days after anthesis), DG (De greening, 16 days after anthesis), WT (White, 20 days after anthesis), IR (Initial red, 23 days after anthesis), PR (Partial red, 25 days after anthesis), and FR (Full red, 28 days after anthesis)] (30). The FR (Full red, 28 days after anthesis) fruit were collected and kept in a 4°C cold room (low temperature, LT) or 37°C incubator (high temperature, HT) for 10 and/or 20 d. The control check (CK) was kept in a 23°C greenhouse (room temperature, RT) at a relative humidity of 70–85%.There are three repetitions for per treatment, and 28 fruits for per replication. Ten fruits were collected on day 3, 7, 10, and/or 20, respectively, and then the seeds and berries were separated with tweezers. Some were used for physical evaluation, and some were frozen in liquid nitrogen for subsequent experiments. Twenty fruits with seeds removed on day 5 were collected for protein expression determination.

### Weight Loss, Firmness, and Color

Fruit weights were recorded, and the percentage weight loss from harvest was calculated. Firmness was measured by a puncture test on each fruit with intact skin using a Force Five pressure tester (Model FDV-30, Wagner Instruments, Greenwich, CT, USA) fitted with a 7.9 mm diameter flathead probe. Surface color of 10 individual fruit per treatment was measured with a hand-held precise color reader (model WR-10; China).

### Anthocyanins Determination

Total anthocyanins were determined using the pH differential method (31). Absorbance was measured in a METASH spectrophotometry (METASH UV-5100) at 520 and 700 nm in buffers at pH 1.0 and 4.5, using A = (A520 – A700)pH 1.0 – (A520 – A700)pH 4.5 with molar extinction coefficients of Cyanidin-3-glucoside (29,600) for strawberry fruit juice. The samples used for anthocyanin components measurement were collected on day 5. Anthocyanin components were determined by liquid chromatography-mass spectrometry (LC-MS), with the peak area used to evaluate the content of each component (31).

### Soluble Sugars, Organic Acids, Hormones, and Cell Wall Components Determinations

0.5 g fruit were ground in liquid nitrogen and mixed with 1.5 ml 80% ethanol, 85°C for 30 min, and centrifuge at 12,000 g for 10 min. Aspirate the supernatant and repeat the above step. Mix the supernatant together and freeze-dry. Fifteen milliliters ultrapure water was used to dissolve lyophilized precipitate and filtered the solution with 0.22 water-filter for the measurement of soluble sugars and organic acids. Soluble sugar and organic acid contents were determined using high-performance liquid chromatography (HPLC) (31, 32).

Hormone contents were determined using the enzyme-linked immunosorbent assay (ELISA) method (32, 33). Protopectin, soluble pectin, cellulose, and hemicellulose were determined using related kits (Solarbio Life Sciences, Beijing, China). The determination of pectin used galacturonic acid as a standard curve, and the absorbance was measured at 530 nm. The determination of cellulose uses glucose as a standard curve. Results were showed as g of per 1,000 g of fresh weight. All measurements were repeated three times.

### Determination of Aroma Compounds

Strawberry fruit (3 g) were ground in liquid nitrogen and mixed with 3 ml saturated NaCl and 2 μl octanol (81.8 mg/L) as an internal standard. Gas chromatography-mass spectrometry (GC-MS) was performed according to the method of Zheng et al. (34). Samples were equilibrated for 10 min at 50°C and then extracted for 30 min with fiber coating divinylbenzene/carboxen/polydimethylsiloxane (DVB/CAR/PDMS) with 50/30-μm thickness. And then, the aroma components were detected with GC system coupled with MS instrument.

### Determination of Amino Acid and Antioxidant Enzyme Activity

Sample of 1 × 10^−5^ kg were added to 0.003 L of 0.02 mol·L^−1^ hydrochloric acid and extracted with sonication for 30 min (minutes). The samples were then extracted overnight at 4°C and centrifuged to obtain the supernatant. One milliliter of the supernatant was mixed with 0.001 L of 4% sulfosalicylic acid and centrifuged to get the supernatant. The supernatant was filtered with a 0.22 μm water-based membrane, and then an automatic amino acid analyzer (L-8900, Hitachi, Japan) was used to determine the amino acid content.

One gram of sample was added to 0.01 L of 0.1 mol·L^−1^ phosphate buffer (pH 7.0), ground into a homogenate, and then centrifuged at 4,000 g for 15 min. The supernatant was used for superoxide dismutase (SOD) and POD determination. Three biological repetitions were conducted. The activity of POD was determined using the guaiacol method (35), and the activity of SOD was determined by the nitro blue tetrazolium (NBT) photochemical reduction method (36).

### Strawberry Fruit Microscopy and Staining

Strawberry fruit paraffin sections were performed as Han et al. (37). Strawberry fruit at different temperatures and times were cut into approximately 5-mm^3^ pieces and fixed in formalin–acetic acid–alcohol (FAA) solution (5% formaldehyde, 5% glacial acetic acid, and 63% ethyl alcohol) at 4°C for 1 week. The strawberry pieces were dehydrated in an ethanol series solution and then embedded in paraffin. Sections were cut to a thickness of 10 μm using a microtome (Leica RM2255, Germany) and stained by 1% (w/v) Toluidine Blue O. After staining, the sections were observed using a microscopy imaging system (Olympus, Tokyo, Japan).

### Protein Extraction and Tandem Mass Tags Proteomic

The sample was ground by liquid nitrogen into a powder. After that, four volumes of phenol extraction buffer (1% protease inhibitor and 1% phosphatase inhibitor) were added to the powder and ultrasonically lysed. An equal volume of Tris was added to equilibrate the phenol and centrifuged at 5,500 g for 10 min at 4°C, and five volumes of 0.1 mol·L^−1^ ammonium acetate/methanol were added to the supernatant for precipitation overnight. The protein precipitate was washed with methanol and acetone. Finally, the protein was redissolved in 8 mol·L^−1^ urea, and the protein concentration was determined with a BCA kit according to the manufacturer's instructions. After trypsin digestion, the peptides were desalted by a Strata X C18 SPE column (Phenomenex) and vacuum-dried. The peptides were reconstituted in 0.5 mol·L^−1^ TEAB and processed according to the manufacturer's protocol for the Tandem Mass Tags (TMT) kit.

### LC-MS/MS Analysis

The peptides were dissolved in the mobile phase A of liquid chromatography [0.1% (v/v) formic acid aqueous solution] and separated using an EASY-nLC 1000 ultra-performance liquid system. Solvent A was an aqueous solution containing 0.1% formic acid and 2% acetonitrile; solvent B was an aqueous solution containing 0.1% formic acid and 90% acetonitrile. The liquid gradient settings were 0–38 min, 9–26% B; 38–52 min, 26–35% B; 52–56 min, 35–80% B; and 56–60 min, 80% B. The flow was maintained at 500 nL/min.

The peptides were subjected to an NSI source followed by tandem mass spectrometry (MS/MS) in a Q Exactive™ Plus (Thermo) coupled online to the ultra-performance liquid chromatograph (UPLC). The electrospray voltage applied was 2.0 kV. Peptide precursor ions and their secondary fragments were detected and analyzed using a high-resolution Orbitrap. The scanning range of the primary mass spectrum was set to 400–1,500 m/z (mass-to-charge ratio), and the scanning resolution was set to 70,000; the scanning range of the secondary mass spectrum was set to a fixed starting point of 100 m/*z*, and the secondary scanning resolution was set to 35,000. Automatic gain control (AGC) was set at 5E4. The signal threshold was set to 38,000 ions/s, the maximum injection time was set to 130 ms, and the dynamic rejection time of the tandem mass spectrometry scan was set to 30 s.

### Bioinformatic Analysis of Proteins

Gene Ontology (GO) annotation proteome was derived from the UniProt-GOA database (http://www.ebi.ac.uk/GOA/). Firstly, converting identified protein ID to UniProt ID and then mapping to GO IDs by protein ID. If some identified proteins were not annotated by UniProt-GOA database, the InterProScan soft would be used to annotated protein's GO functional based on protein sequence alignment method. Then proteins were classified by Gene Ontology annotation based on three categories: biological process, cellular component and molecular function. Kyoto Encyclopedia of Genes and Genomes (KEGG) database was used to annotate protein pathway. Firstly, using KEGG online service tools KAAS to annotated protein's KEGG database description. Then mapping the annotation result on the KEGG pathway database using KEGG online service tools KEGG mapper. All differentially expressed protein database accession or sequence were searched against the STRING database version 10.1 for protein-protein interactions. Only interactions between the proteins belonging to the searched data set were selected, thereby excluding external candidates. STRING defines a metric called “confidence score” to define interaction confidence; we fetched all interactions that had a confidence score ≥0.7 (high confidence).

### RNA Extraction and Quantitative Real-Time (qRT)-PCR

Total RNA was extracted from the strawberry using Quick RNA Isolation Kit (Hua Yue Yang, Beijing China). The cDNA was reverse transcribed according to HifairTM II 1st Strand cDNA Synthesis SuperMix Kit for qRT-PCR (gDNA digester plus) (Yeasen, Shanghai, China). Specific primers were designed using online software Primer 3 Plus (http://primer3.ut.ee/; Supplementary Table 1). For different transcripts, NCBI Gene (https://www.ncbi.nlm.nih.gov) was used for comparing the difference of sequences between different transcripts, and then designed specific primers using the different part of the transcript sequence. TaKaRa SYBR Premix Ex Taq™ II (Takara, Dalian, China) was used to perform qPCR on ABI prism 7900 Real-Time PCR system (Applied Biosystems, USA). The thermal cycles were set as follows: 95°C for 2 min for pre-incubation, 40 cycles of 95°C for 10 s and 58°C for 30 s for amplification. The relative gene expression levels were calculated by the 2^−Δ*ΔCt*^ method. The strawberry gene *Actin* was used as the reference.

### Gene Transient Expression in Strawberry

Transient expression in strawberry was conducted according to Zheng et al. (38). The constructed vectors *FaP1-GFP, FaP2-GFP*, and *FaP3-GFP* were transfected into Agrobacterium (strain EHA105), following which the strawberry fruit were infected with pCAMBIA-1302 as CK at the large green stage (14 days after anthesis). Seven days later, the anthocyanin content, soluble sugar and organic acid content, and cell wall component content were determined. The antioxidant enzyme activities of POD and SOD were determined, and volatile organic compounds were determined using -GC-MS.

### Statistical Analysis

The statistics were analyzed using statistical analysis of variance (ANOVA) SPSS statistics 17.0 (SPSS Inc., Chicago, IL, USA), TB tools v1.072 (39), and Origin Pro 9 (Origin Inc., Northampton, MA, USA). All experiments were performed at least three replicates. Experimental data were presented as means ± standard deviation (SD). Duncan *post-hoc* test (*P* <0.05) was performed to test the existence of statistical differences between different treatments.

## Results

### Effect of Temperature Regimes on Strawberry Fruit Quality

**Figure 1:**
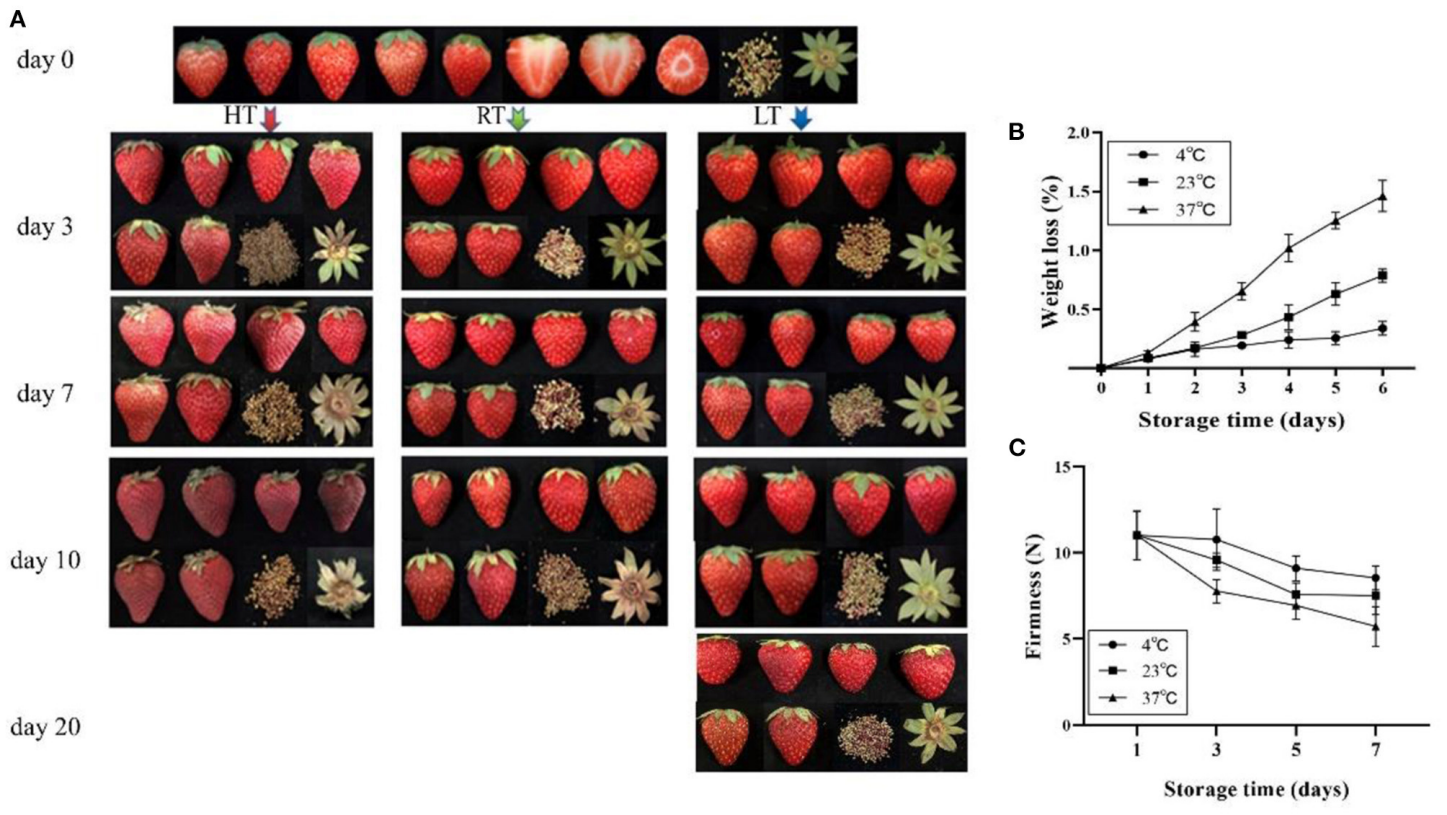
Overall quality of strawberry at 4, 23, and 37°C. **(A)** Appearance of fruit; **(B)** weight loss; and **(C)** firmness change of fruit. The fruit color developed into a dark red and lost its glossiness over time and temperature. Fruit stored at 4°C maintained a better quality and firmness, but 37°C accelerated fruit weight loss 1 day later. LT, RT, and HT represent the storage temperature of 4, 23, and 37°C, respectively. Each value represents the mean of three replicates. Error bar stands for standard deviation (SD) and date are expressed as means ± SD (*n* = 3, three replications).

To determine the the physical index changes of strawberry in response to different temperatures, fruit color, anthocyanin content, fruit sugar and organic acid content, amino acid content, and plant hormone content were measured on day 3, 7, and 10 at 4, 23, and 37°C storage temperature treatments. The results showed that the fruit color developed into a dark red and lost its glossiness with prolonged storage time, and an increase in temperature resulted in more severe discoloration (Figure 1A).

#### Fruit Weight Loss, Firmness, and Color

Weight loss from the berries increased over time (Figure 1B) and was affected by storage temperature. Among the three temperature, weight losses were similar on day 1, but by day 5 were 0.24 and 0.63% at 4 and 23°C, respectively; and by day 4 were 1.05% at 37°C. Firmness was affected by temperature and decreased over time (Figure 1C). At 4°C, firmness decreased by 2.21% for the first 3 days, while decreased by 29.39% at 37°C. Lightness (L^*^ value) and chroma (C^*^ value) were changes over time (Table 1). At 4°C, lightness of the strawberry fruit was not affected by storage time but significantly declined with higher storage temperatures. Chroma increased slightly at 4°C, but decreased at higher temperature (23 and 37°C).

**Table 1:** Lightness and chroma value of strawberry in fruit stored for up to 1, 3, 5, and 7 days at 4, 23, and 37°C.

**Storage time**	L*			C*		
	**4**°**C**	**23**°**C**	**37**°**C**	**4**°**C**	**23**°**C**	**37**°**C**
Day 1	41.18 ± 1.01			54.54 ± 4.81		
Day 3	39.00 ± 1.51^a^	35.10 ± 1.12^b^	28.33 ± 1.17^c^	53.97 ± 7.34	54.96 ± 2.22	48.20 ± 6.20
Day 5	37.55 ± 1.72^a^	32.20 ± 2.72^b^	26.80 ± 2.30^c^	55.73 ± 1.14^a^	52.79 ± 2.57^a^	44.52 ± 3.09^b^
Day 7	37.62 ± 1.74^a^	28.24 ± 1.12^b^	24.30 ± 0.72^c^	60.83 ± 4.79^a^	49.19 ± 2.37^b^	36.39 ± 2.48^c^

L*, lightness; C*, chroma. Error bar stands for standard deviation (SD) and date are expressed as means ± SD. Duncan test was used to significance analysis (P < 0.05).

a, b, c *Represent the significance level between different treatments at the same time (P < 0.05)*.

#### Anthocyanins

**Figure 2:**
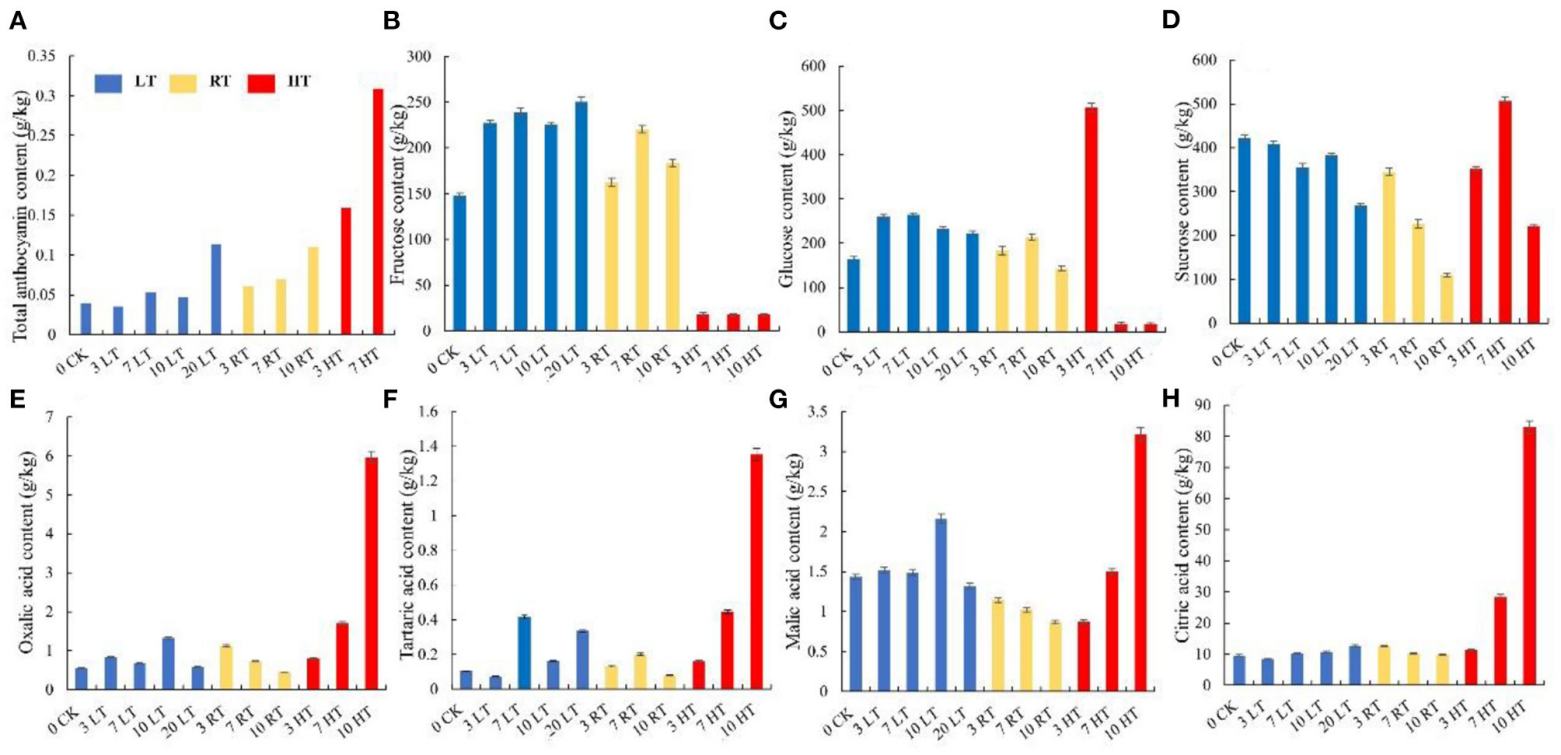
Total anthocyanin, sugars, and organic acids content in strawberry at 4, 23, and 37°C. Total anthocyanin content was higher at higher temperature **(A)**; higher soluble sugars contents and lower organic acids were measured at 4°C, while the situation was opposite 37°C **(B–H)**. LT, RT, and HT represent the storage temperature of 4, 23, and 37°C, respectively. Each value represents the mean of three replicates. Error bar stands for standard deviation (SD) and date are expressed as means ± SD.

Furthermore, higher temperatures resulted in greater anthocyanin accumulation (Figure 2A), among which, more cyanidin 3-galactoside, cyanidin, pelargonidin, cyaniding 3,5-diglucoside, and luteolin were accumulated under high temperature (Table 2; Supplementary Table 2). Soluble sugar and organic acid of strawberry were affected by temperature and days. Patterns of change were similar between 4 and 23°C, but more fructose, glucose, and sucrose were accumulated at 4°C (Figures 2B–D). Overall, high temperature promoted the accumulation of organic acid (Figures 2E–H).

**Table 2:** Anthocyanin content in strawberry berry and seed at 4, 23, and 37°C.

**Components (g·kg^**−1**^ DW)**	**Storage temperature**					
	**LT berry**	**RT berry**	**HT berry**	**LT seed**	**RT seed**	**HT seed**
Cyanidin 3-galactoside	1.87	7.82	19.81	1.66	1.62	0.37
Procyanidin B4	0.50	0.54	0.77	0.5	0.44	0.24
Procyanidin B2	0.33	0.51	1.02	0.37	0.37	0.39
Cyanidin	0.23	0.55	2.07	0.27	0.45	0.24
Rutin	0.11	0.39	0.75	0.12	0.14	0.11
Cyaniding 3,5-diglucoside	0.11	0.39	0.75	0.12	0.14	0.11
Luteolin	0.01	0.01	0.64	0.02	0.01	0.01
Quercetin	0.01	0.00	0.01	0.01	0.01	0.01
Pelargonidin	10.85	22.11	40.16	Nd	Nd	Nd
Kaempferol	0.03	0.03	0.05	0.05	0.05	0.06
Isorhamnetin	0.01	0.00	0.01	0.01	0.01	0.01
Petunidin	0.02	0.07	0.02	0.09	0.09	0.10

*Pelargonidin was the main anthocyanin, which was only detected in berry. Thirty-seven degree Celsius induced the more anthocyanins contents in berry, such as cyanidin 3-galactoside, cyanidin, pelargonidin, cyaniding 3,5-diglucoside, and luteolin. Lower anthocyanins contents were detected at 4°C. LT, RT, and HT represent the storage temperature of 4, 23, and 37°C, respectively. Nd represents that the component was not detected*.

#### Amino Acid and Hormones

The content of amino acid was also affected by storage time and temperature (Supplementary Figure 1). Generally, temperature has a greater influence on the amino acids in strawberry berry. Among the 17 amino acids, the proportion of alanine was increased at higher temperature. Leucine and isoleucine were higher at 37°C than at 4 and 23°C. In addition, threonine content increased at 4°C, while γ-aminobutyric acid increased at 37°C. IAA, ABA, methyl jasmonate, zeatin, and brassinosteroid are important hormones in regulating plant growth and development and stress resistance (40). Our results showed that five hormones were all induced by 37°C treatment, and more IAA was detected in the seed and less ABA was detected in the berry at 4°C (Supplementary Figure 2).

#### Volatile Organic Compounds

To determine the effect of temperature on aroma, the component of volatile organic compounds in strawberry were measured. In strawberries, alcohols, aldehydes, esters, alkanes, acids, aromatic benzenoids, ketone, and alkenes were the main components. Among them, esters were markedly affected by storage time and temperature (Supplementary Figure 3; Supplementary Table 3). Hexanoic acid ethyl ester, hexanoic acid methyl ester, and ethyl acetate were the compounds most affected by high temperature. Their levels increased rapidly in the first 3 days at 37°C, then declined. Octanoic acid ethyl ester showed increased with time at 4 and 23°C, while at 37°C, its level was rapidly increased and then declined. Acetic acid hexyl ester decreased with time in all three temperatures. The content of alcohols was higher at low temperature than at high temperature. Linalool content showed a low level at 37°C. Aldehydes and acids were less in strawberry fruit and among the detected aldehydes, 2-hexenal and hexanal were decreased with time and almost not detected at 23 and 37°C 3 days later. Ketones showed low level at 4°C, but significantly increased with storage temperature. The content of 4-methoxy-2,5-dimethyl-3(2H)-furanone was low at 4°C, while at 37°C, it accounted for 44.26% on day 10.

#### Cell Structure and Cell Wall Composition

**Figure 3:**
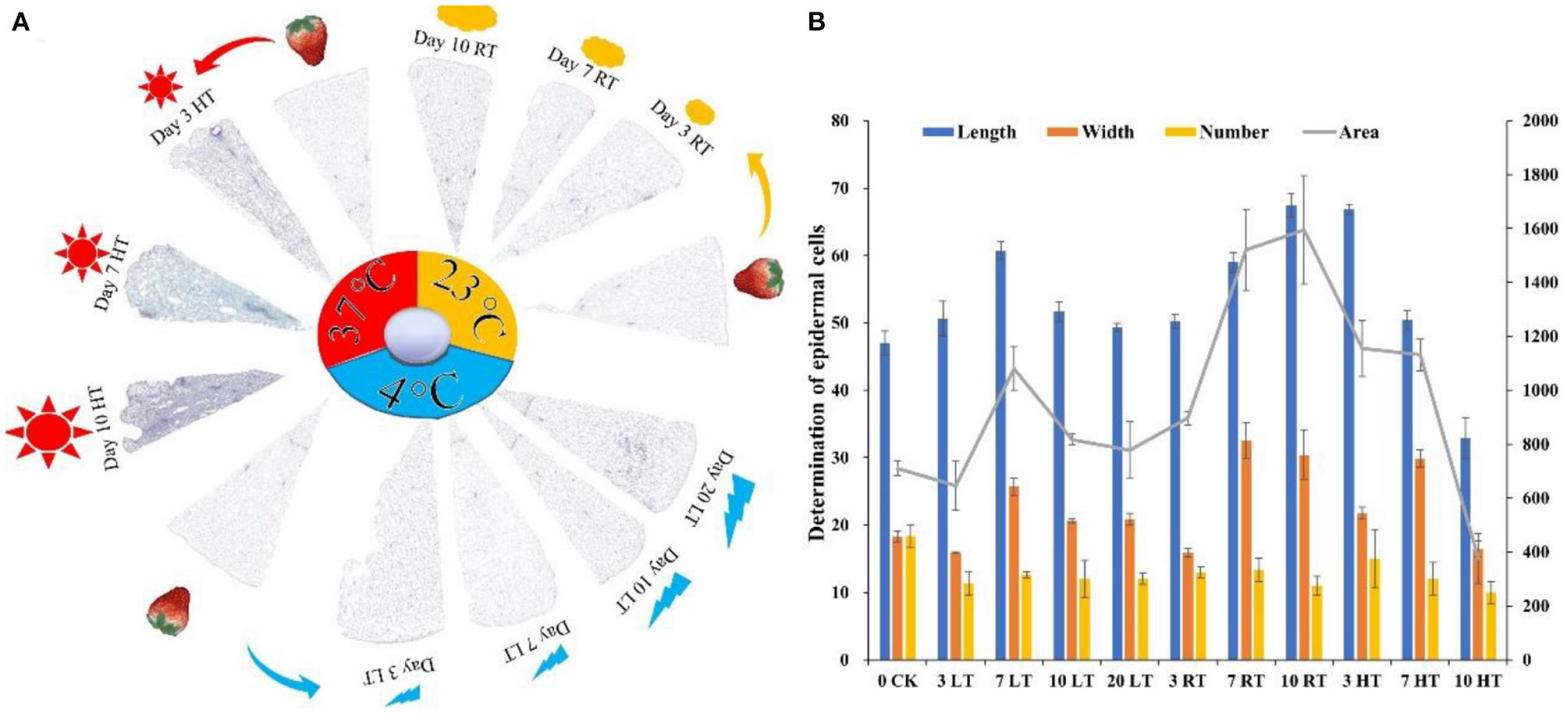
Paraffin section **(A)** and determination of epidermal cells **(B)** of strawberry structure at 4, 23, and 37°C. Four degree Celsius contained a good tissue structure, while destroyed at 37°C **(A)**; the number, length, and width of epidermal cells were deceased at 37°C. The tissue fills the entire imaging field of view to ensure that the background light of each photo is consistent (*n* = 3). LT, RT, and HT represent the storage temperature of 4, 23, and 37°C, respectively. Each value represents the mean of three replicates. Error bar stands for standard deviation (SD) and date are expressed as means ± SD.

To further investigate the effects of temperature on cell tissue, paraffin sections, and cell wall composition were measured. As shown in Figure 3A, cell structure was destroyed under high temperature from the day 3, and the higher the temperature, the severe the damage. Meanwhile, the change in the width and area of the epidermal cells under the same field of view was measured, which showed that the biggest change was at HT and then at RT (Figure 3B). The number of epidermal cells at low temperature remained basically unchanged between day 3 and 7, while at RT and HT, the number of cells was increased by 4 and 6.3 times, respectively (Figure 3B). The cell wall composition was greatly affected by high temperature, showed high content from day 3 (Supplementary Figure 4). The content of hemicellulose, cellulose and soluble pectin remained similar for the first 7 days. The content of protopectin was higher at 23°C than 4°C from day 3.

### Proteomics Profile in Response to Different Temperatures

**Figure 4:**
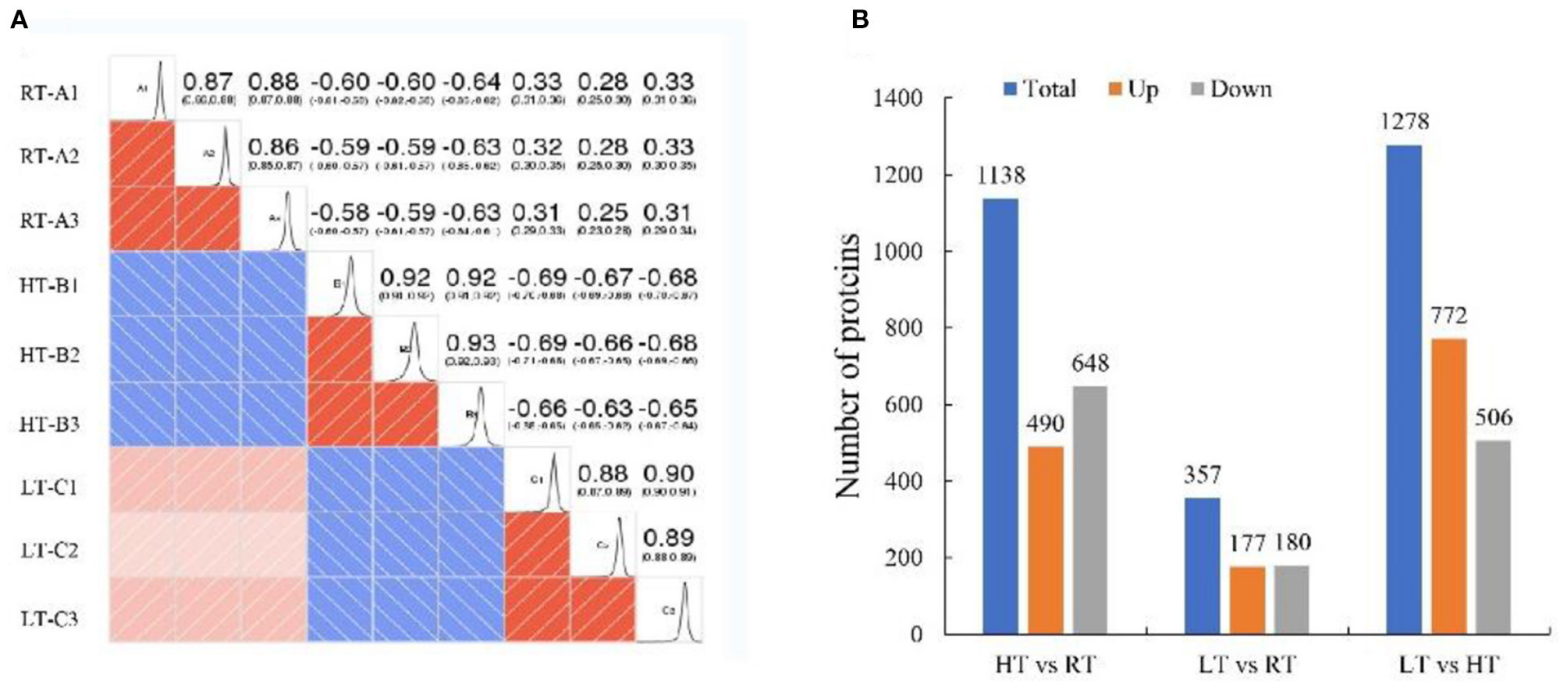
Proteomic analysis of samples at 4, 23, and 37°C. **(A)** Pearson correlation coefficient. **(B)** Number of differentially expressed proteins. LT, RT, and HT represent the storage temperature of 4, 23, and 37°C, respectively.

We used TMT proteomics to identify and quantify the induced proteins under various storage temperature conditions. The data analysis indicated that the total identified proteins were 6,657 and 5,595 of them were quantifiable. The number of differentially expressed proteins (DEPs) was summarized in a heatmap based on Pearson's correlation coefficient (Figure 4A). A total of 1,138 DEPs was identified in HT vs. RT, including 490 upregulated and 648 downregulated DEPs; LT vs. RT consisted of 177 upregulated and 180 downregulated DEPs; and LT vs. HT had 772 upregulated and 506 downregulated DEPs (Figure 4B).

#### Functional Enrichment Analysis of DEPs

**Figure 5:**
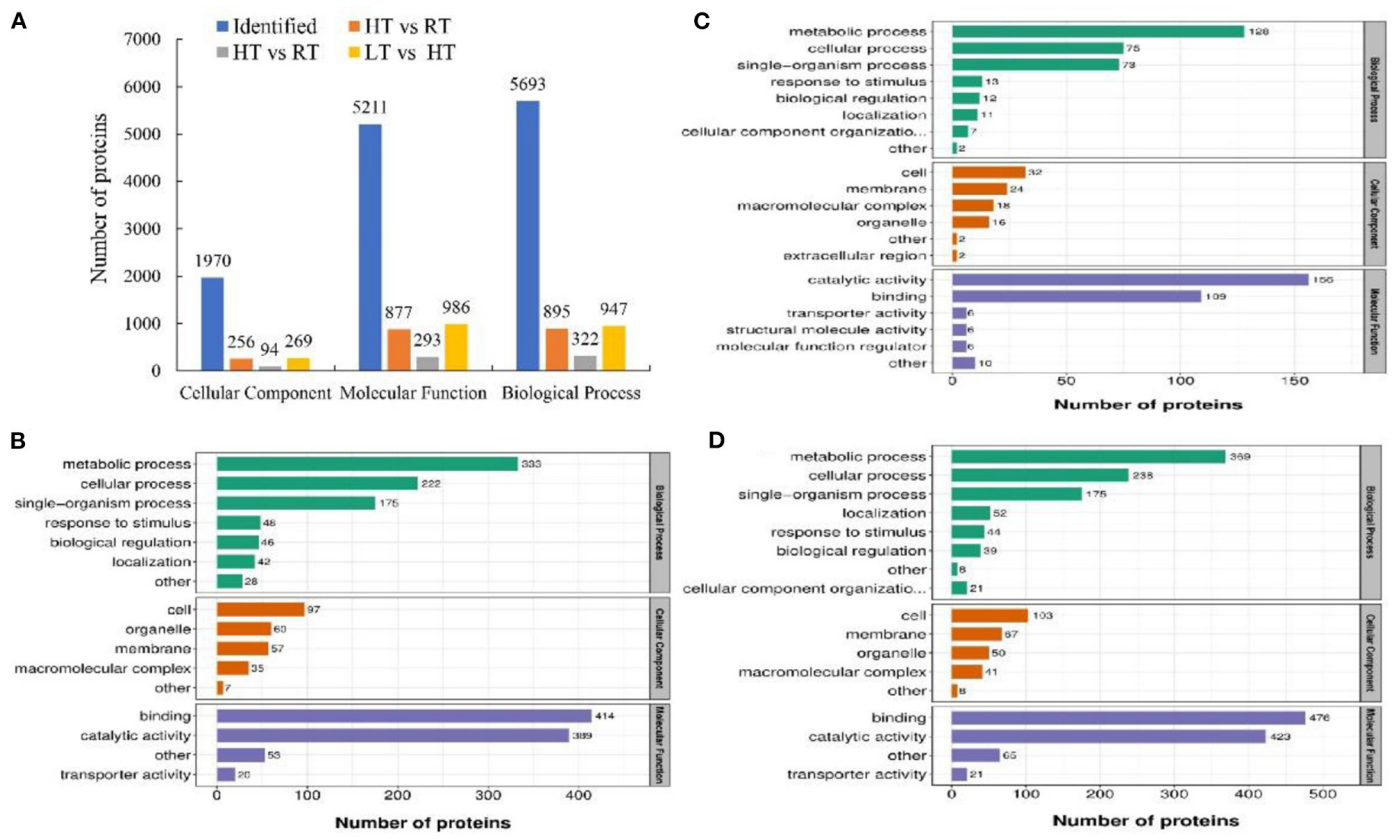
Number of enriched proteins and GO classification involved in specific biological processes in different comparisons at 4, 23, and 37°C. **(A)** Number of identified proteins and significantly enriched proteins with *P* < 0.05 involved in three categories including biological process, cellular component and molecular function. **(B–D)** Number of different GO terms in three categories. In **(B–D)** green columns represented the categorization of biological process, orange columns represented the categorization of cellular component, and blue columns represented the categorization of molecular function. **(B)** HT vs. RT; C: LT vs. RT; D: LT vs. HT. LT, RT, and HT represent the storage temperature of 4, 23, and 37°C, respectively.

The associations of proteins with specific biological processes were identified and were divided into three major groups, including cellular component (CC, 1970), molecular function (MF, 5211), and biological process (BP, 5693) (Figure 5A). In HT vs. RT, the detected enriched proteins included CC (256), MF (877), and BP (895). The most enriched proteins in MF were binding (414) followed by catalytic activity (389); in CC, cell (97) and organelle (60) were highly enriched, and in the BP category, metabolic process showed the highest number of proteins (333) followed by cellular process (222) (Figures 5A,B). In LT vs. RT, MF, BP, and CC were enriched with 293, 322, and 94 proteins, respectively, and most of the proteins were enriched in biological process (Figures 5A,C). In LT vs. HT, the enriched proteins included 269 in CC, 986 in MF, and 947 in BP (Figures 5A,D).

To investigate the role of proteins in metabolic pathways, we analyzed the DEPs according to KEGG enrichment analysis (Supplementary Figure 5). Glutathione metabolism and cutin, suberin, and wax biosynthesis pathways were upregulated in HT vs. RT, while phenylpropanoid biosynthesis, flavonoid biosynthesis, and starch and sucrose metabolism downregulated. Compared with the RT, photosynthesis and photosynthesis-antenna proteins were upregulated under LT condition, while glycolysis/gluconeogenesis, and biosynthesis of secondary metabolites pathways were downregulated. Additionally, In the LT vs. HT, photosynthesis, phenylpropanoid biosynthesis, and starch and sucrose metabolism were upregulated, while glutathione metabolism, and ascorbate and aldarate metabolism were downregulated. In addition, the protein interaction networks for all listed samples were determined, and protein–protein interaction (PPI) maps were generated with the closely interacting proteins (Supplementary Figure 6; Supplementary Table 4). A great number of proteins interacted with heat shock protein.

#### Analysis of Proteins in Phenylpropanoid Biosynthetic Pathway

**Figure 6:**
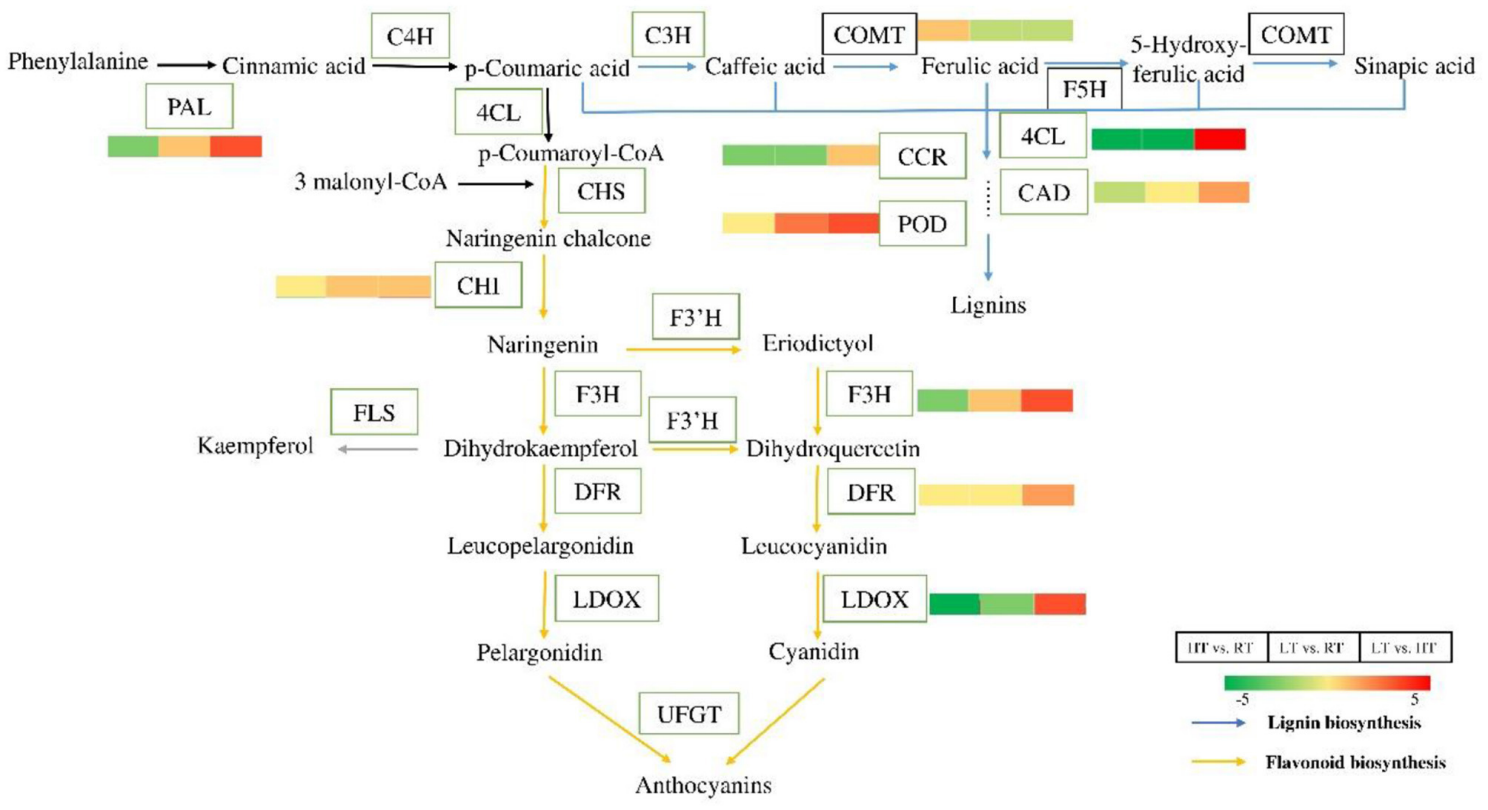
Differential proteins in the process of phenylpropane biosynthesis at 4, 23, and 37°C. Flavonoids and lignin are important secondary metabolite of the phenylpropane pathway. Almost all the proteins were downregulated in flavonoids and lignin biosynthesis at 4 and 37°C. But the related proteins were upregulated at 4°C compared to at 37°C. The color bars represent the number of up-regulated or down-regulated protein. The greener the color, the more the down-regulated proteins, and the redder the color, the more the up-regulated proteins. PAL, phenylalanine ammonia-lyase; C4H, coumarate 3-hydroxylase; 4CL, 4-coumarate—CoA ligase; CHS, chalcone synthase; CHI, chalcone—flavonone isomerase; F3H, flavanone 3-hydroxylase; F3′H, flavonoid-3′-hydroxylase; DFR, dihydroflavonol 4-reductase; LDOX, leucoanthocyanidin dioxygenase; UFGT, UDP-glycose flavonoid glycosyltransferase; FLS, flavonol synthase; C3H, coumarate 3-hydroxylase; COMT, caffeic acid 3-O-methyltransferase; F5H, ferulate-5-hydroxylase; CCR, cinnamoyl-CoA reductase; CAD, cinnamyl alcohol dehydrogenase; POD, peroxidase.

Phenylpropanoid biosynthetic is one of the important ways for plants to produce secondary metabolites, such as flavonoids, lignin (41). Twenty-five DEPs involved in phenylpropanoid biosynthesis under different condition were identified (Figure 6; Table 3; Supplementary Tables 5–7). Compared with at 23°C condition, 23 and 17 DEPs were identified at 37 and 4°C, respectively. Most of them were downregulated, including phenylalanine ammonia-lyase (PAL), 4-coumarate—CoA ligase (4CL), dihydroflavonol 4-reductase, leucoanthocyanidin dioxygenase, and cinnamoyl-CoA reductase, while only two POD proteins were upregulated at 4°C. Eighteen DEPs were found in LT vs. HT, and all of them were upregulated except for two caffeic acid 3-O-methyltransferase (COMT).

**Table 3:** Differentially expressed proteins involved in phenylpropane metabolism processes in strawberry at 4, 23, and 37°C.

**Protein accession**	**Protein description**	**Ratio**		
		**HT vs. RT**	**LT vs. RT**	**LT vs. HT**
FANhyb_icon00000880_a.1.g00001.1	Chalcone—flavonone isomerase 3	0.606	–	–
FANhyb_icon00001777_a.1.g00001.1	Naringenin,2-oxoglutarate 3-dioxygenase	0.391	–	1.94
FANhyb_icon00005664_a.1.g00001.1	Naringenin,2-oxoglutarate 3-dioxygenase	0.411	–	1.925
FANhyb_rscf00004797.1.g00001.1	Naringenin,2-oxoglutarate 3-dioxygenase	0.54	–	1.714
FANhyb_rscf00000482.1.g00003.1	Bifunctional dihydroflavonol 4-reductase/flavanone 4-reductase	0.335	0.66	1.982
FANhyb_rscf00005879.1.g00001.1	Leucoanthocyanidin dioxygenase-like	0.193	0.45	2.344
FANhyb_icon00024324_a.1.g00001.1	Leucoanthocyanidin dioxygenase-like	0.307	0.21	–
FANhyb_icon00046410_a.1.g00001.1	Leucoanthocyanidin dioxygenase-like	0.377	0.35	–
FANhyb_icon00025903_a.1.g00001.1	Leucoanthocyanidin dioxygenase	0.417	–	1.965
FANhyb_icon00007826_a.1.g00001.1	Leucoanthocyanidin dioxygenase	0.448	–	2.008
FANhyb_rscf00002856.1.g00001.1	4-coumarate—CoA ligase 2-like	0.324	0.55	1.7
FANhyb_icon16011461_o.1.g00001.1	4-coumarate—CoA ligase-like 7	0.344	0.59	1.706
FANhyb_icon19669573_s.1.g00001.1	4-coumarate—CoA ligase-like 7	0.218	0.44	2.009
FANhyb_icon18385291_s.1.g00001.1	4-coumarate—CoA ligase-like 7	0.303	0.55	1.826
FANhyb_icon00012602_a.1.g00001.1	4-coumarate—CoA ligase-like 7	0.305	0.5	1.648
FANhyb_icon00005046_a.1.g00001.1	Phenylalanine ammonia-lyase 1	0.369	–	1.964
FANhyb_rscf00000079.1.g00001.1	Phenylalanine ammonia-lyase 1	0.317	–	2.424
FANhyb_rscf00000868.1.g00006.1	Phenylalanine ammonia-lyase 1	0.349	–	2.066
FANhyb_icon00034475_a.1.g00001.1	Caffeic acid 3-O-methyltransferase	–	0.55	0.589
FANhyb_rscf00000219.1.g00012.1	Caffeic acid 3-O-methyltransferase	–	0.56	0.593
FANhyb_rscf00005355.1.g00001.1	Cinnamoyl-CoA reductase 1-like	0.284	0.41	–
FANhyb_icon00042818_a.1.g00001.1	Cinnamoyl-CoA reductase 1-like	0.37	0.43	–
FANhyb_rscf00000152.1.g00015.1	Cinnamoyl-CoA reductase 1-like	0.334	0.4	–
FANhyb_rscf00001371.1.g00002.1	Cinnamyl alcohol dehydrogenase 1	0.328	–	2.911
FANhyb_rscf00000027.1.g00015.1	Cinnamyl alcohol dehydrogenase 6	0.297	0.25	–

*Most proteins identified in phenylpropane metabolism pathway were involved in flavonoids and lignin biosynthesis. Almost all proteins were downregulated at 4 and 37°C compared to at 23°C, including phenylalanine ammonia-lyase, 4-coumarate—CoA ligase, flavanone 3-hydroxylase, leucoanthocyanidin dioxygenase, and caffeic acid 3-O-methyltransferase. But these proteins were upregulated at 4°C compared to at 37°C*.

#### Analysis of ROS-Related DEPs

The redox-related DEPs under different temperatures are presented in the Supplementary Table 4. At 37°C, the expression of TPR repeat-containing thioredoxin TDX and one POD protein was induced. Only a single catalase (CAT) protein was identified and upregulated by the high temperature treatment. High temperatures triggered antioxidant enzymes as well as AsA-GSHcycle enzymes. Monodehydroascorbate reductase (MDAR) and three ascorbate peroxidase (APX) proteins were identified, and all four proteins were upregulated. Seven glutathione S-transferase (GST) proteins were found, six of which were upregulated, and a single GST protein was down-regulated (Table 4). In addition, there were nine DEPs (three upregulated and six downregulated) that were modulated at 4°C, including two thioredoxin-like proteins (one upregulated and one downregulated), one downregulated phospholipid hydroperoxide glutathione peroxidase, two upregulated PODs, one downregulated APX, and three downregulated GST proteins. In LT vs. HT, 21 DEPs (3 upregulated and 18 downregulated) were identified. The downregulated proteins included three thioredoxin-like proteins, one CAT, one MDAR and six APX and six GST proteins. The three upregulated proteins were POD protein (Table 4).

**Table 4:** Differentially expressed proteins involved in ROS scavenging system at 4, 23, and 37°C.

**Protein accession**	**Protein description**	**Ratio**		
		**HT vs. RT**	**LT vs. RT**	**LT vs. HT**
FANhyb_rscf00002393.1.g00001.1	Catalase isozyme 1	1.558	–	0.588
FANhyb_icon00012053_a.1.g00001.1	Glutathione S-transferase	1.507	0.653	0.433
FANhyb_rscf00007218.1.g00001.1	Glutathione S-transferase	1.699	–	0.426
FANhyb_icon00020921_a.1.g00001.1	Glutathione S-transferase	3.903	0.639	0.164
FANhyb_rscf00004731.1.g00002.1	Glutathione S-transferase	8.691	–	0.108
FANhyb_icon00039178_a.1.g00001.1	Glutathione S-transferase	–	–	0.573
FANhyb_rscf00000671.1.g00005.1	Glutathione S-transferase 3	1.718	–	–
FANhyb_icon00002658_a.1.g00001.1	Glutathione S-transferase L3-like isoform X1	0.633	0.62	–
FANhyb_rscf00000493.1.g00004.1	Glutathione S-transferase T1	1.903	–	0.58
FANhyb_icon00010153_a.1.g00001.1	L-ascorbate peroxidase	–	–	0.58
FANhyb_icon00016963_a.1.g00001.1	L-ascorbate peroxidase	–	–	0.58
FANhyb_icon00004634_a.1.g00001.1	L-ascorbate peroxidase	–	–	0.61
FANhyb_rscf00006913.1.g00001.1	L-ascorbate peroxidase 2	1.698	0.637	0.375
FANhyb_icon00013544_a.1.g00001.1	L-ascorbate peroxidase 2	2.021	–	0.332
FANhyb_rscf00001324.1.g00002.1	L-ascorbate peroxidase 3	1.539	–	0.478
FANhyb_rscf00003756.1.g00001.1	Monodehydroascorbate reductase	1.525	–	0.637
FANhyb_rscf00000251.1.g00015.1	Peroxidase 16-like	0.472	1.774	3.762
FANhyb_rscf00000806.1.g00003.1	Peroxidase 17-like	–	–	2.049
FANhyb_icon00005967_a.1.g00002.1	Peroxidase 63	–	1.727	2.559
FANhyb_icon00010632_a.1.g00001.1	Phospholipid hydroperoxide glutathione peroxidase	0.587	–	1.856
FANhyb_rscf00003733.1.g00002.1	Phospholipid hydroperoxide glutathione peroxidase	0.609	0.645	–
FANhyb_icon00056001_a.1.g00001.1	Phospholipid hydroperoxide glutathione peroxidase	0.652	–	–
FANhyb_icon16313727_o.1.g00001.1	Thioredoxin H-type	–	–	0.517
FANhyb_icon00007759_a.1.g00001.1	Thioredoxin H-type	–	–	0.576
FANhyb_rscf00000027.1.g00001.1	Thioredoxin-like protein CDSP32	–	1.767	–
FANhyb_rscf00000665.1.g00003.1	Thioredoxin-like protein YLS8	–	0.651	0.143
FANhyb_rscf00002203.1.g00001.1	TPR repeat-containing thioredoxin TDX	1.506	–	–
FANhyb_icon00027113_a.1.g00001.1	TPR repeat-containing thioredoxin TDX	1.755	–	–

*POD-related proteins were upregulated under 4°C storage condition. More proteins were induced by 37°C, such as catalase, thioredoxin, L-ascorbate peroxidase, and monodehydroascorbate reductase. The ratio is differential expression changes of protein between different temperature treatments. A ratio >1.5 represents up-regulation, while a ratio <1/1.5 represents down-regulation*.

#### Analysis of the HSP Family

HSPs are stress-responsive proteins, that can be expressed in large quantities under adversity. High temperature induced the expression of HSPs in the different comparisons, including high molecular weight HSPs and low molecular weight HSPs (Table 5; Supplementary Tables 5–7). All of the DEPs were upregulated in HT vs. RT, except heat shock cognate 70 kDa protein-like (FANhyb_rscf00001174.1g00004.1). In contrast, the same proteins showed the opposite expression pattern (upregulated) in LT vs. HT (Table 5; Supplementary Table 6). However, the differentially expressed protein in LT vs. RT was not found, which indicated that 4°C treatment could not cause any damage to the strawberry fruit.

**Table 5:** Information of heat shock-related differentially expressed proteins at 4, 23, and 37°C.

**Protein accession**	**Protein description**	**Ratio**	
		**HT vs. RT**	**LT vs. HT**
FANhyb_rscf00001174.1.g00004.1	Heat shock cognate 70 kDa protein-like	0.469	2.168
FANhyb_rscf00003942.1.g00001.1	Stromal 70 kDa heat shock-related protein, chloroplastic	1.871	0.504
FANhyb_rscf00000523.1.g00005.1	Heat shock 70 kDa protein, mitochondrial	2.132	0.417
FANhyb_rscf00000249.1.g00002.1	Heat shock 70 kDa protein 8	2.258	0.435
FANhyb_rscf00004349.1.g00001.1	Heat shock cognate 70 kDa protein 2-like	2.625	0.43
FANhyb_rscf00001325.1.g00004.1	Heat shock cognate 70 kDa protein 2	3.013	0.278
FANhyb_rscf00000060.1.g00021.1	Heat shock cognate protein 70-1	6.953	0.148
FANhyb_rscf00000129.1.g00011.1	Heat shock protein 83	3.84	0.328
FANhyb_rscf00000129.1.g00010.1	Heat shock protein 83-like	2.965	0.485
FANhyb_rscf00002208.1.g00001.1	Heat shock protein 83	1.934	0.555
FANhyb_rscf00001100.1.g00002.1	Heat shock protein 90-1	2.08	0.419
FANhyb_icon00006799_a.1.g00001.1	Class I heat shock protein-like	8.069	0.157
FANhyb_icon00018260_a.1.g00001.1	Class I heat shock protein-like	10.074	0.083
FANhyb_icon00042861_a.1.g00001.1	23.6 kDa heat shock protein	1.646	–
FANhyb_icon13773961_s.1.g00001.1	23.6 kDa heat shock protein	4.32	0.224
FANhyb_rscf00000056.1.g00014.1	18.1 kDa class I heat shock protein-like	5.225	0.194
FANhyb_icon00011084_a.1.g00001.1	26.5 kDa heat shock protein	5.952	0.116
FANhyb_icon00002668_a.1.g00001.1	Heat shock 22 kDa protein, mitochondrial isoform X2	6.268	0.174
FANhyb_rscf00000148.1.g00013.1	17.9 kDa class II heat shock protein-like	6.272	0.206
FANhyb_rscf00000151.1.g00006.1	Small heat shock protein	6.46	0.177
FANhyb_rscf00001249.1.g00001.1	17.4 kDa class III heat shock protein	7.597	0.131
FANhyb_rscf00002246.1.g00002.1	18.1 kDa class I heat shock protein-like	10.344	0.13
FANhyb_rscf00004595.1.g00002.1	17.8 kDa class I heat shock protein-like	10.73	0.086
FANhyb_rscf00003182.1.g00002.1	18.1 kDa class I heat shock protein-like	11.604	0.125
FANhyb_icon00013124_a.1.g00001.1	15.7 kDa heat shock protein	11.621	0.081
FANhyb_icon00027613_a.1.g00001.1	26.5 kDa heat shock protein	13.996	0.076
FANhyb_icon15018869_s.1.g00001.1	18.1 kDa class I heat shock	20.134	0.079
FANhyb_rscf00000024.1.g00031.1	Small heat shock protein	9.349	0.096
FANhyb_icon00008156_a.1.g00001.1	Heat shock 22 kDa protein	9.1	0.114

*Twenty-nine heat shock proteins were identified and all of them increased at 37°C*.

#### Analysis of Cell Wall Degrading Enzymes

In response to high temperature at 37°C, two polygalacturonase (PG), three beta-galactosidase (β-Gal) isoforms, and one pectinesterase (PE) were downregulated compared with 23°C. Five xyloglucan endotransglucosylase/hydrolase (XTH) proteins were identified, and three of them were downregulated. Expansin-A10 was downregulated (Table 6; Supplementary Tables 5–7). Furthermore, PG expression was induced at 4°C and four β-Gal proteins were found as compared to 23°C. Among the four β-Gal proteins, three of them were upregulated, while one was downregulated. Four PE proteins were found, and two of them were downregulated, while the other two were upregulated. Two XTH DEPs were identified, and one of them was downregulated, while the other one was upregulated. Expansin-A10 showed down-regulated (Table 6). In the comparison of LT vs. HT, one PG protein and five β-Gal proteins were upregulated, and four PE proteins were identified, two of which were downregulated, while the other two were upregulated. Three XTH proteins and one expansin (EXP) protein were found, all of which were upregulated (Table 6).

**Table 6:** Differentially expressed proteins involved in the cell wall degradation in strawberry at 4, 23, and 37°C.

**Protein accession**	**Protein description**	**Ratio**		
		**HT vs. RT**	**LT vs. RT**	**LT vs. HT**
FANhyb_rscf00000714.1.g00004.1	Exopolygalacturonase-like	0.209	0.338	6.079
FANhyb_rscf00000239.1.g00005.1	Polygalacturonase	0.328	–	–
FANhyb_rscf00000038.1.g00011.1	Beta-galactosidase 16	0.248	0.504	2.034
FANhyb_rscf00000073.1.g00021.1	Beta-galactosidase 3	0.623	–	1.54
FANhyb_rscf00003668.1.g00001.1	Beta-galactosidase 9	0.666	–	–
FANhyb_rscf00000181.1.g00012.1	Beta-galactosidase isoform X5	–	1.522	1.902
FANhyb_rscf00006987.1.g00001.1	Beta-galactosidase 1	–	1.623	–
FANhyb_rscf00000052.1.g00019.1	Beta-galactosidase 5	–	2.022	2.471
FANhyb_icon19873497_s.1.g00001.1	Beta-galactosidase 8-like	–	–	1.683
FANhyb_rscf00000265.1.g00001.1	Pectinesterase 3	0.361	1.822	5.052
FANhyb_icon19600037_s.1.g00001.1	Pectinesterase 3	0.65	3.055	4.7
FANhyb_icon00013246_a.1.g00001.1	Pectinesterase-like	–	0.618	0.613
FANhyb_icon00034303_a.1.g00001.1	Pectinesterase-like	–	0.62	0.637
FANhyb_icon00001439_a.1.g00001.1	Probable xyloglucan endotransglucosylase/hydrolase protein B	0.309	–	3.969
FANhyb_icon00001861_a.1.g00001.1	Xyloglucan endotransglucosylase/hydrolase protein 23	0.535	0.624	–
FANhyb_icon00011859_a.1.g00001.1	Probable xyloglucan endotransglucosylase/hydrolase protein 23	0.653	–	1.958
FANhyb_icon00012405_a.1.g00001.1	Xyloglucan endotransglucosylase/hydrolase protein 6	1.733	–	–
FANhyb_icon00002288_a.1.g00001.1	Xyloglucan endotransglucosylase/hydrolase protein 6	2.33	1.995	–
FANhyb_rscf00000020.1.g00003.1	Xyloglucan endotransglucosylase/hydrolase protein 8	–	–	1.846
FANhyb_rscf00000201.1.g00004.1	Expansin-A10	0.134	0.543	4.039

*The proteins of PG, PE, β-Gal, and EXP were downregulated at 37°C compared to at 23°C. The proteins of PG, EXP, and some PE were downregulated at 4°C compared to at 23°C. Meanwhile, all the identified proteins, except PE, were upregulated at 4°C compared to 37°C. PG, polygalacturonase; PE, pectinesterase; β-Gal, beta-galactosidase; XTH, xyloglucan endotransglucosylase/hydrolase; EXP, expansin*.

#### Verification of DEPs via qPCR

The proteins involved in anthocyanins metabolic pathway and cell wall degradation, including *FaPAL, FaCHS, FaF3H, FaCHI, FaDFR, FaANS, FaPG, FaPE, FaCEL, FaPL, Fa*β*-GAL*, and *FaEXP*, were further confirmed by qRT-PCR. As shown in Supplementary Figure 7, the expression levels of some genes related to anthocyanin synthesis were upregulated on day 3 and then downregulated on day 7. Most of the genes expressed at 4°C were decreased for the first 7 days, and then increased on day 10, ultimately decreasing on day 20. Under the storage temperature of 23°C, a large percentage of genes was decreased with the increasing storage time. In addition, some gene expression levels were enhanced on day 3 after 37°C treatment, such as *FaCHS, FaCHI2, FaF3H, FaDFR2*, and *FaGST*. Furthermore, the expression level of cell wall degradation related genes was decreased at 4°C during the first 7 days and then increased on day 10 (Supplementary Figure 7). For example, the expression level of the *FaPG, FaPL, FaCEL, Fa*β*-GAL*, and *FaEXP2* genes showed a similar trend. At high temperature, the genes expression levels of *FaPG, FaPL, FaCEL*, and *FaEXP* were downregulated with storage time, and *FaPE* maintained a higher expression level on day 7. Meanwhile, *FaCEL, FaEXP1*, and *FaEXP2* were kept higher expression level on day 3. *FaPE, FaXEP1*, and *FaEXP5* were upregulated at 23°C, and *Fa*β*-GAL* maintained a higher expression level during storage (Supplementary Figure 7). Overall, the trend of gene expression was consistent with the results of the TMT data.

### Function Analysis of Proteins Selected From TMT

**Figure 7:**
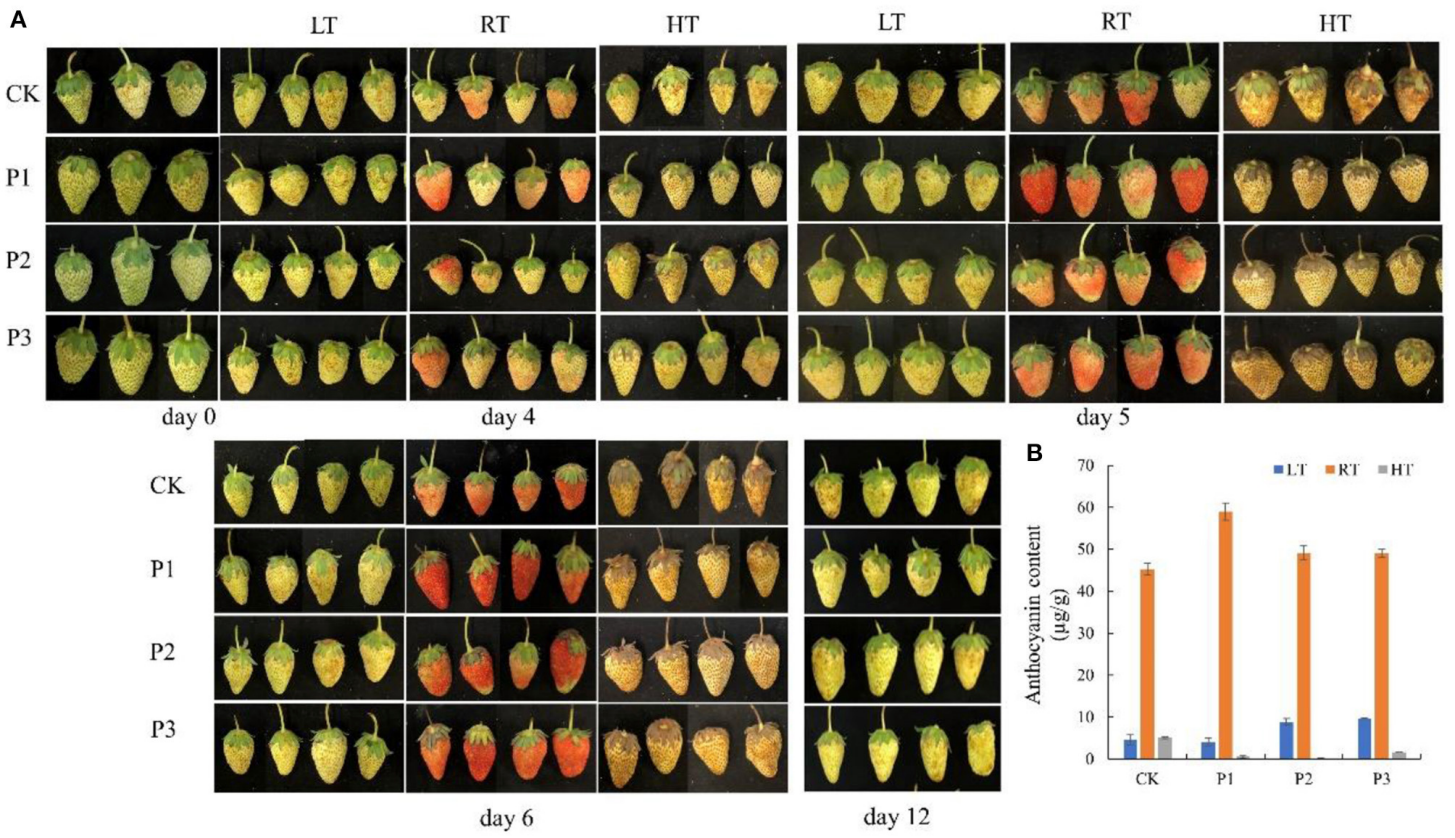
Overexpression of proteins in strawberry at 4, 23, and 37°C. All three proteins significantly promoted fruit coloring at 23°C from the 4th day after overexpression and delayed fruit browning at 4°C **(A)**; total anthocyanin content was significantly increased at 23°C after protein overexpression, and P2 and P3 increased the anthocyanin content at 4°C **(B)**. P1, sugar phosphate/phosphate translocator; P2, 1-aminocyclopropane-1-carboxylate oxidase; P3, aquaporin PIP2-2. LT, RT, and HT represent the storage temperature of 4, 23, and 37°C, respectively. Each value represents the mean of three replicates. Error bar stands for standard deviation (SD) and date are expressed as means ± SD.

**Figure 8:**
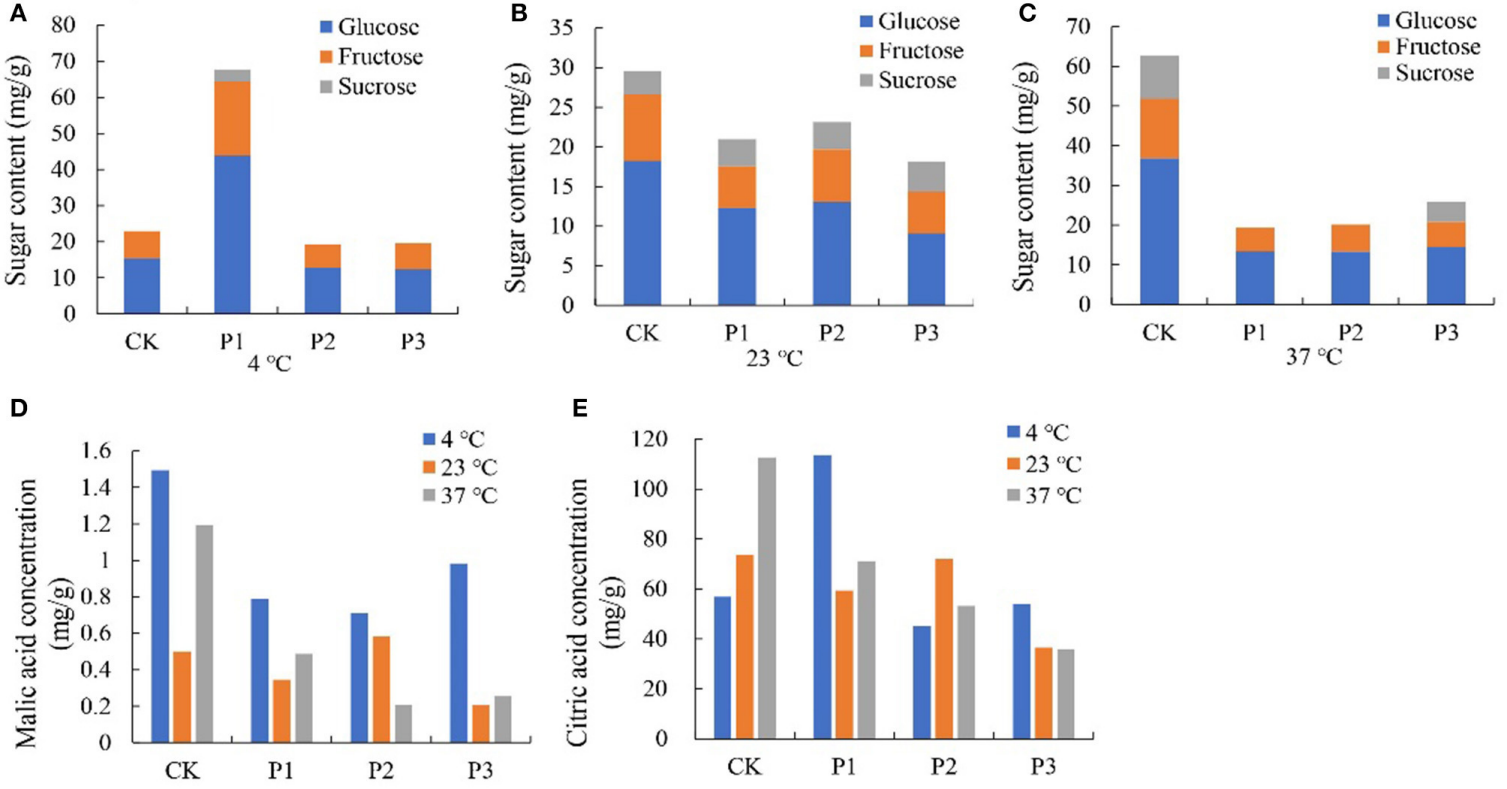
Effect of different overexpressed proteins on sugars and organic acids content in strawberry at 4°C, 23°C and 37°C. **(A–C)** The sugar content of strawberry at different storage temperatures; **(D,E)** The malic and citric acid content of strawberry at different storage temperatures. P1 protein promoted sugar and citric acid accumulation at 4°C, but decreased the sugar and organic acid content at 23°C and 37°C. Both the P2 and P3 proteins decreased the sugar content and organic acid content at three temperatures. P1, P2 and P3 reprented proteins of sugar phosphate/phosphate translocator; 1-aminocyclopropane-1-carboxylate oxidase and aquaporin PIP2-2, respectively.

To investigate the function of proteins under temperature stress, we selected three proteins from TMT, including sugar phosphate/phosphate translocator (P1; GenBank Accession No. XM_004291415.2), 1-aminocyclopropane-1-carboxylate oxidase (P2; XM_004304031.2), and aquaporin PIP2-2 (P3; XM_004291529.2), and performed a BLAST search in the NCBI library. The results showed that all three proteins increased fruit color at 23°C from the 4th day after overexpression and delayed fruit browning at 4°C (Figure 7A). The total anthocyanin content was increased at 23°C after protein overexpression; however, it decreased at 37°C (Figure 7B). The P1 protein promoted sugar and citric acid accumulation at 4°C, but decreased the sugar and organic acid contents at 23 and 37°C; and both the P2 and P3 proteins decreased the sugar content and organic acid content at different temperatures (Figure 8).

#### Protein Overexpression Altered Aromatic Components

At 4°C storage temperature, 2-hexenal, diazirine, hexanal, and trichloromethane were the main aromatic components, and acetic acid ethenyl ester was only induced by P3, which accounted for 64.89% of the total volatiles. Esters and aldehydes were the main aromatic at 23°C, including 2-hexenal, 2-hexen-1-ol, acetate, (Z)-, acetic acid, methyl ester, and hexanal, but the main aromatic components were not changed. Furthermore, the P1 and P3 proteins also induced more aromatic components at 4 and 23°C, respectively, while the P2 protein induced more aromatic components at 37°C. Moreover, the P1 and P2 proteins induced greater trichloromethane, and toluene content. In addition, trichloromethane, and hexanal were increased by P3. 1-Hexanol and ethyl acetate were not detected in the strawberries under high temperature treatment (Supplementary Figure 8; Supplementary Table 8).

#### Protein Overexpression Altered Cell Wall Component Content and Antioxidant Enzyme Activities

Cell wall component contents and antioxidant enzyme activities were assessed to further evaluate the function of P1, P2, and P3 proteins on fruit quality. The results showed that all three proteins increased the hemicellulose content at 23°C but decreased it at 37°C in comparison with the control (Supplementary Figure 9A). The P1 and P3 proteins decreased the cellulose content at 4 and 37°C, whereas the P3 protein increased the cellulose content at 23°C (Supplementary Figure 9B). In addition, the soluble pectin content was decreased after protein overexpression, and the protopectin content at 37°C was increased by P2 (Supplementary Figures 9C,D). Furthermore, the three proteins increased SOD activity at 4 and 37°C, while P3 protein activity was increased at 23°C. All three proteins increased the POD activity at 4 and 23°C, but decreased its activity at 37°C (Supplementary Figures 9E,F).

#### Protein Overexpression Ripening Associated Gene Expression

The expression of *FaPAL* and *FaCHS* was upregulated at low temperatures, while the expression of *FaCHI2, FaANS, FaUFGT*, and *FaGST* expression was downregulated (Supplementary Figure 10). In addition, *FaCHI1, FaDFR1*, and *FaDFR2* were upregulated by P1, and *FaF3H* was upregulated by P3. *FaPE* and *FaEXP* were downregulated by all three proteins, except for *FaPE* and *FaXEP1*, which were upregulated by P1. The expression of *FaPG, FaPL*, and *FaCEL* was decreased by the P1 protein, but increased by the P2 protein. The P3 protein had no effect on *FaPG* and *FaCEL*, but did upregulated *Fa*β*-GAL*.

Most genes, including *FaCHS, FaCHI, FaF3H*, and *FaGST* were downregulated with the overexpression of the three proteins at 23°C compared with the control (Supplementary Figure 10). P1 upregulated *FaPAL, FaDFR*, and *FaUFGT* expression, and only *FaDFR1* was upregulated by the P2 protein. In addition, *FaPE, FaPL, Fa*β*-GAL*, and *FaEXP2* were downregulated by all three proteins, while *FaEXP1* and *FaEXP5* were upregulated. *FaPG* and *FaCEL* were also downregulated by the P2 and P3 proteins.

The gene expression results at 37°C were similar to the results of the three proteins (Supplementary Figure 10). *FaPLA, FaCHS, FaDFR1, FaUFGT*, and *FaGST* were upregulated, while *FaCHI1, FaF3H, FaDFR2*, and *FaANS* were downregulated by the three proteins. Most cell wall-related genes were upregulated by the three proteins, such as *FaPG, FaPL, FaCEL*, and *FaEXP*, and only *FaPE* was downregulated. As for *Fa*β*-GAL*, the expression was upregulated by P1, which was opposite to that of the P2 protein.

## Discussion

The appearance of fruit is the most important index for evaluating strawberry quality. Previous studies focus on the effect of low temperature on fruit quality (5, 13, 42). However, few reports have examined the effect of long-term high temperature storage on fruit quality and the response mechanism. Therefore, this study examined the influence of 4, 23, and 37°C storage temperatures to fruit quality and explored the mechanism of fruit response to different temperatures at the proteome level.

### Storage Temperature Affected the Quality of Strawberry After Harvest

Storage at 4°C resulted in better visual quality than at 23°C, which was better than at 37°C (Figure 1A). An important result of loss of visual quality was the onset of water loss (Figure 1B). These results were similar to those of Shin et al. (22) who found that lower temperature maintained a better fruit quality and decreased the rate of fruit water loss. The fruit, stored at 4 and 23°C, remained relatively stable red color, but at 37°C, color changed to dark red (Figure 1A). The change of anthocyanin content was consistent with fruit color (Figure 2A). Higher storage temperature result in greater anthocyanin accumulation (5, 13, 20).

Decrease of firmness and loss of fruit peel lightness are common processes during the senescence of strawberry fruit (43). In our study, firmness and lightness kept a higher level at 4°C but significantly decreased at 37°C (Figure 1C; Table 1). Harker et al. (44) found that firmness of fruit is related to a number of cellular characteristics, including adhesion between neighboring cells, cell fragility, and internal turgor pressure. The maintenance of hardness at low temperature might be related to a better cell structure (Figure 3).

The special aroma of strawberry fruit is composed of a variety of volatile organic compounds in a certain proportion. The different content of one or several aroma components may lead to different odors. Over 360 volatile organic compounds have been identified from strawberries (45), but only a few volatiles (primarily methyl and ethyl esters) were the main contributors to strawberry aroma (5). In our study, higher level esters were induced by higher storage temperature (46), while lower level aldehydes were detected at the same time (Supplementary Figure 1; Supplementary Table 3). The amino acid metabolism pathway is an important way promoting the synthesis of aroma. In our study, the increased proportion of alanine, leucine, and isoleucine might this be the main reason for the formation of esters compounds at higher temperature (47).

Sugars and organic acids are important indexes for evaluating strawberry fruit quality and flavor (22). Good strawberry flavor is resulted in high sugar and relatively high acid content. Study have shown that glucose and fructose were the major sugars in strawberry fruit, which accounted for more than 65% of total soluble solids (48). In our study, fructose and glucose contents on day 7 and 10 were markedly lower at 37°C than lower temperature, while acid contents were higher 3 days later at 37°C (Figure 2). Increased respiration rates are likely to be the cause of a lower sugar content at elevated temperatures (5). In addition, sugars and acids, as osmotic regulators, also participated in the stress resistance process of plants (49).

### Effect of Storage Temperature on Fruit Cell Wall Degradation and Phenylalanine Metabolism

The protein involved in fruit softening is mainly by inducing cell wall polysaccharide degradation or cell wall loosening (50, 51). PG is an enzyme involved in degradation of the pectic fraction of cell walls, but its action is not sufficient to promote fruit softening alone (51, 52). Other proteins, such as EXPs, may be involved in the process by inducing loosening of fruit cell walls, they have variety differences in strawberries (52). Despite the complexity of fruit softening and the presumably high number of proteins involved, most proteins identified in our study were downregulated at 37°C (Table 6). The identification of these proteins involved the loss of firmness could be related to the expression of related genes under different stress times.

Phenylpropane metabolism is an important way to produce plant secondary metabolites, which contribute to plant responses toward biotic and abiotic stimuli (53, 54). Flavonoids are one of the important branches of plant phenylpropane metabolism, which play an important role in the interaction between plants and environment (54, 55). CHS and F3H have been characterized as rate-limiting enzymes in flavonoid pathway, and studies have shown that they were strongly upregulated in strawberry at 2-d cold treatment (4). Though these proteins were identified downregulated in response to cold and heat stress, related gene expression increased on day 3 and then decreased, suggesting that these proteins were induced under short-term stress. LDOX is a key enzyme at the end of the anthocyanin synthesis pathway (55). In our study, the abundance of LDOX protein decreased at 37°C, but the content of anthocyanin increased in response to heat stress. Though strawberry is a non-climactic fruit, previous study has confirmed that respiration functioned up-stream of the ethylene-dependent signaling in the regulation of anthocyanin synthesis and high temperature can enhance the respiratory rate and ethylene production (56). We speculate that, on the one hand, high temperature could enhance proteins expression by increasing respiration and ethylene production. However, on the other hand, high temperature could also directly reduce protein expression in the fruit. Additional studies are needed to clarify this supposition.

### Temperature Treatment Induced the Antioxidant System and HSPs in Strawberry Fruit

Plant contain components of enzymic and non-enzymic antioxidants which play an important role in regulating level of reactive oxygen species (ROS) in response to abiotic stress (57). In this study, POD was up-regulated during LT storage, while down-regulated during HT storage (Figure 6). PODs are enzymes that typically catalyze peroxide. Dangcham et al. (58) found that POD activity could induced by low temperature in mangosteen pericarp during storage. In addition, POD is required in the final step of lignin biosynthesis, and lignin content is associated with pericarp hardening (58, 59). The higher hardness of strawberries at 4°C, in our study, might be related to high POD activity (Figure 1C). Here, we found that the abundance of catalase isozyme 1 was significantly increased at HT compared to other storage temperatures (Table 4). CAT mainly removes hydrogen peroxide (H_2_O_2_) produced in the process of mitochondrial electron transport, β-fatty acid oxidation, and photorespiration to prevent active oxygen free radicals from harming plants (59, 60). Here, the higher abundance of CAT protein at HT storage found in this study suggested an important role in for this enzyme in cellular redox sensing under HT stress (Table 4).

Ascorbate-glutathione (AsA-GSH) cycle is one of the most important plant antioxidant systems (25). APX is one of the most important enzymes associated with the AsA-GSH cycle, and its over-expression in tomato confers tolerance to chilling stresses (61). Interestingly, the abundance of APX protein, in this study, was increased at HT, while decreased at LT. These results were consistent with Ergin et al. (62), who found that the activities of APX and CAT in both heat tolerant and heat sensitive cultivars increased with high temperatures. It is suggested that APX played a crucial role in HT stress.

HSPs, as molecular chaperones, have an important part in maintaining protein stability under stress conditions, such as drought, heat, heavy metal, and oxygen deprivation (62, 63). As shown from the TMT analysis, HSP70, HSP83, HSP90-1, and small HSPs were strongly upregulated by 37°C (Table 5). It has been reported that plant such as potato, Arabidopsis, and strawberry begin to synthesize HSP when they exposed to heat stress (64, 65). Although some studies have shown that some HSPs can be induced by cold, in this study, HSPs protein were only identified at HT. The difference of these proteins indicated that the accumulation of HSP might be related to cultivars and treatment temperatures (4).

### Selected P1, P2, and P3 Proteins Improved Fruit Stress Resistance

P1 belongs to the triose-phosphate transporter (TPT) family, which plays a crucial role in photosynthesis (63, 66). A previous study also confirmed that TPT is essential for the survival to *Plasmodium berghei* (67). In this study, the P1 protein enhanced the concentration of sugar, citric acid, and protopectin at 4°C, as well as POD and SOD activities, which suggested that P1 played an important role in improving the antioxidant activity of the fruit and delaying the reduction of fruit flavors. However, the contents of sugars, organic acids, and cell wall components were reduced by the P1 protein under 37°C, which indicated that high temperature had a negative effect on maintaining fruit quality substances.

P2 is one of the key enzymes in ethylene synthesis (68), and a previous study confirmed that the expression of the P2 gene was upregulated in plants subjected to environmental stress (69). In our study, the contents of sugars and organic acids were decreased by P2 at different storage temperatures, while the protopectin content and SOD activity were promoted. Meantime, the POD activity at lower temperature was also induced (Figure 8; Supplementary Figure 9). These results suggested that the P2 protein had a positive response to different temperatures.

P3 is a subclass of the smallest aquaporin family located on the endoplasmic reticulum membrane (70). Xu et al. (71) found that *TaTIP2;2* may be a negative regulator of abiotic stress by the heterologous expression of *TaTIP2;2* in *Arabidopsis thaliana*. Our study results showed that the P3 protein decreased sugar, organic acid, and cellulose contents at 37°C, and the sugar and organic acids were similar between at 4°C and the control, while 4°C enhanced the content of hemicellulose and POD activity (Figure 8; Supplementary Figure 9). These results indicated that the P3 protein has a negative effect on fruit quality at high temperature, but plays an important role in low temperature stress.

In summary, the data presented in this study indicated that storage temperature significantly affected strawberry anthocyanin, aroma compounds, cell structure, sugars, organic acids, and overall quality. New details information is presented on the effect of storage temperature on strawberry anthocyanins, aroma, and antioxidant capacity from the perspective of proteomics. These resulted suggested that the overall quality was better maintained at 4°C, but ester aroma compounds and antioxidant capacity were enhanced at higher temperature (23 and 37°C). Though the proteins involved in anthocyanin synthesis were downregulated, anthocyanin content was enhanced.

## Data Availability Statement

The mass spectrometry proteomics data have been deposited to the ProteomeXchange Consortium via the PRIDE partner repository with the dataset identifier PXD030184.

## Author Contributions

TZ and JL conducted sample treatment, data analysis, and drafted the manuscript. TD and ZS helped with data analysis. HJ participated in the design of the study. TP revised the manuscript. HJ and JF designed and coordinated this study and revised the manuscript. All authors have read and approved the final manuscript.

## Funding

This study was supported by the National Key Research and Development Project (No. 2018YFD1000200), National Natural Science Foundation of China (Nos. 31872938 and 31872047), Jiangsu Excellent Youth Fund Project (No. BK20180076), Jiangsu Natural Science Foundation (No. iBK20201176), Natural Science Research Project of Colleges and Universities in Jiangsu (No. 20KJD210001), and Jiangsu Independent Innovation of Agricultural Science and Technology (No. CX(19)3088).

## Conflict of Interest

The authors declare that the research was conducted in the absence of any commercial or financial relationships that could be construed as a potential conflict of interest.

## Publisher's Note

All claims expressed in this article are solely those of the authors and do not necessarily represent those of their affiliated organizations, or those of the publisher, the editors and the reviewers. Any product that may be evaluated in this article, or claim that may be made by its manufacturer, is not guaranteed or endorsed by the publisher.

## Supplementary Material

Supplementary Figure 1 Amino acid content in seed and berry of strawberry at 4, 23, and 37°C.

Supplementary Figure 2 Hormone concentration in seed and berry at 4, 23, and 37°C.

Supplementary Figure 3 Strawberry aroma components mass spectrum at 4, 23, and 37°C.

Supplementary Figure 4 Cell wall component content of strawberry at 4, 23, and 37°C.

Supplementary Figure 5 Differentially expressed proteins (DEPs) in KEGG pathways at 4, 23, and 37°C.

Supplementary Figure 6 Protein interaction network in different metabolism pathways at 4, 23, and 37°C.

Supplementary Figure 7 Relative gene expression level in strawberry at 4, 23, and 37°C.

Supplementary Figure 8 Strawberry aroma components mass spectrum after proteins overexpression at 4, 23, and 37°C.

Supplementary Figure 9 Cell wall component content and antioxidant enzyme activity after proteins overexpression at 4, 23, and 37°C.

Supplementary Figure 10 Effect of proteins overexpression on relative gene expression level at 4, 23, and 37°C.

Supplementary Table 1 Primers used for qRT-PCR on strawberry.

Supplementary Table 2 Chromatogram of anthocyanin content in strawberry berry and seed.

Supplementary Table 3 Aroma content in strawberry under different storage temperature.

Supplementary Table 4 Differentially expressed protein involved in interaction network.

Supplementary Table 5 Differentially expressed proteins in HT vs. RT.

Supplementary Table 6 Differentially expressed proteins in LT vs. RT.

Supplementary Table 7 Differentially expressed proteins in LT vs. HT.

Supplementary Table 8 Aroma content after protein overexpression.

## Abbreviations

APX: ascorbate peroxidase
CAT: catalase
EXP: expansin
GST: glutathione S-transferase
HSPs: heat shock proteins
HT: high temperature (37°C)
LT: low temperature (4°C)
PAL: phenylalanine ammonia-lyase
P1: sugar phosphate/phosphate translocator
P2: 1-aminocyclopropane-1-carboxylate oxidase
P3: aquaporin PIP2-2
PG: polygalacturonase
RT: room temperature (23°C)
4CL: 4-Coumarate—CoA ligase
XTH: xyloglucan endotransglucosylase/hydrolase
β-Gal: beta-galactosidase.
