# Supplementary material for: Strawberry Proteome Responses to Controlled Hot and Cold Stress Partly Mimic Post-harvest Storage Temperature Effects on Fruit Quality
Source: Front Nutr. 2022 Feb 15;8:812666. doi: 10.3389/fnut.2021.812666 (PMC8887963; doi:10.3389/fnut.2021.812666)

**Figure S1** **Amino acid content in seed and berry of strawberry at 4 °C, 23 °C and 37 °C.** LT, RT and HT represent the storage temperature of 4 °C, 23 °C and 37 °C, respectively. Each value represents the mean of three replicates.


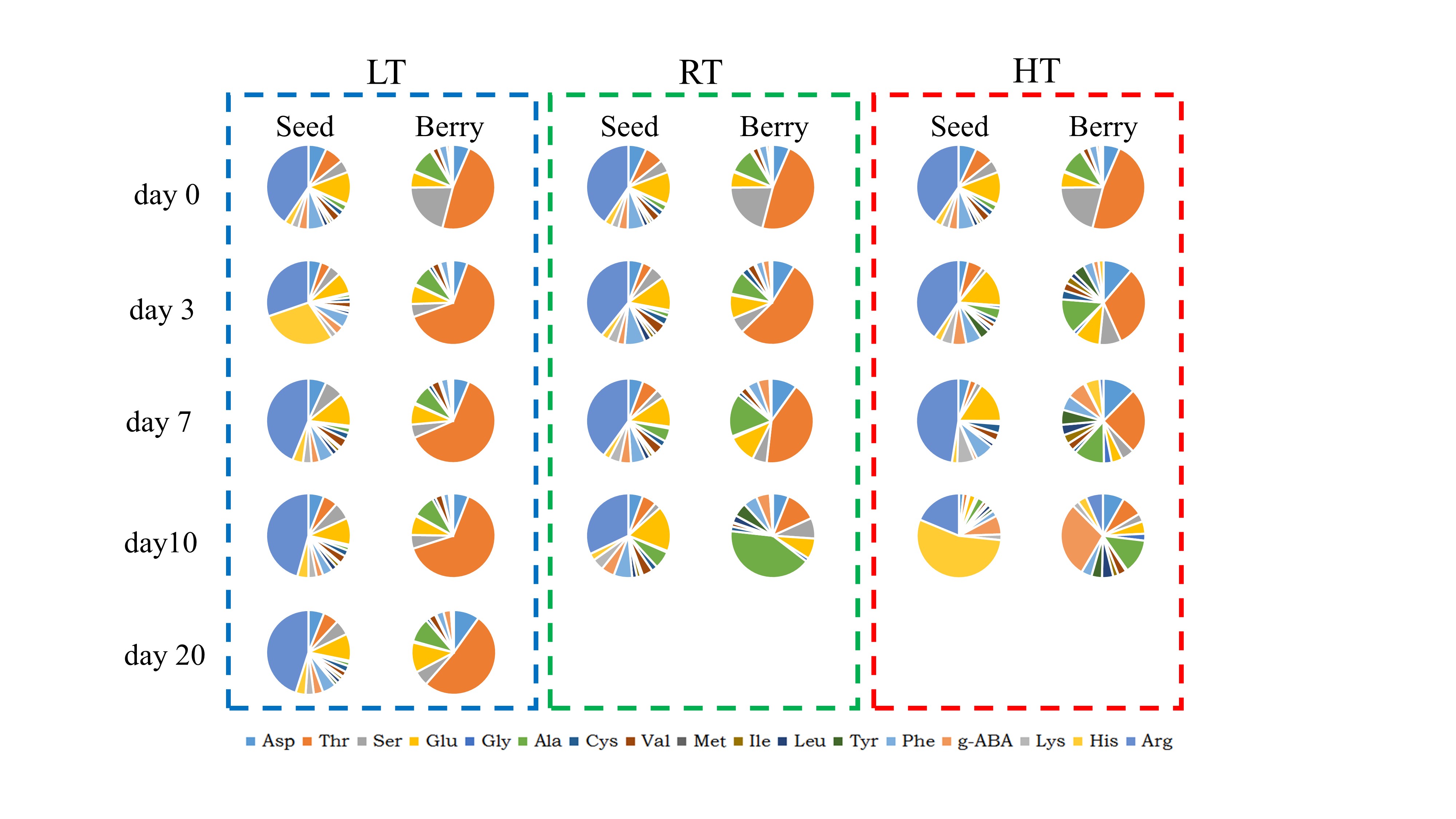


**Figure S2 Hormone concentration in seed and berry at 4 °C, 23 °C and 37 °C.** LT, RT and HT represent the storage temperature of 4 °C, 23 °C and 37 °C, respectively. Each value represents the mean of three replicates. Error bar stands for standard deviation (SD) and date are expressed as means ± SD.


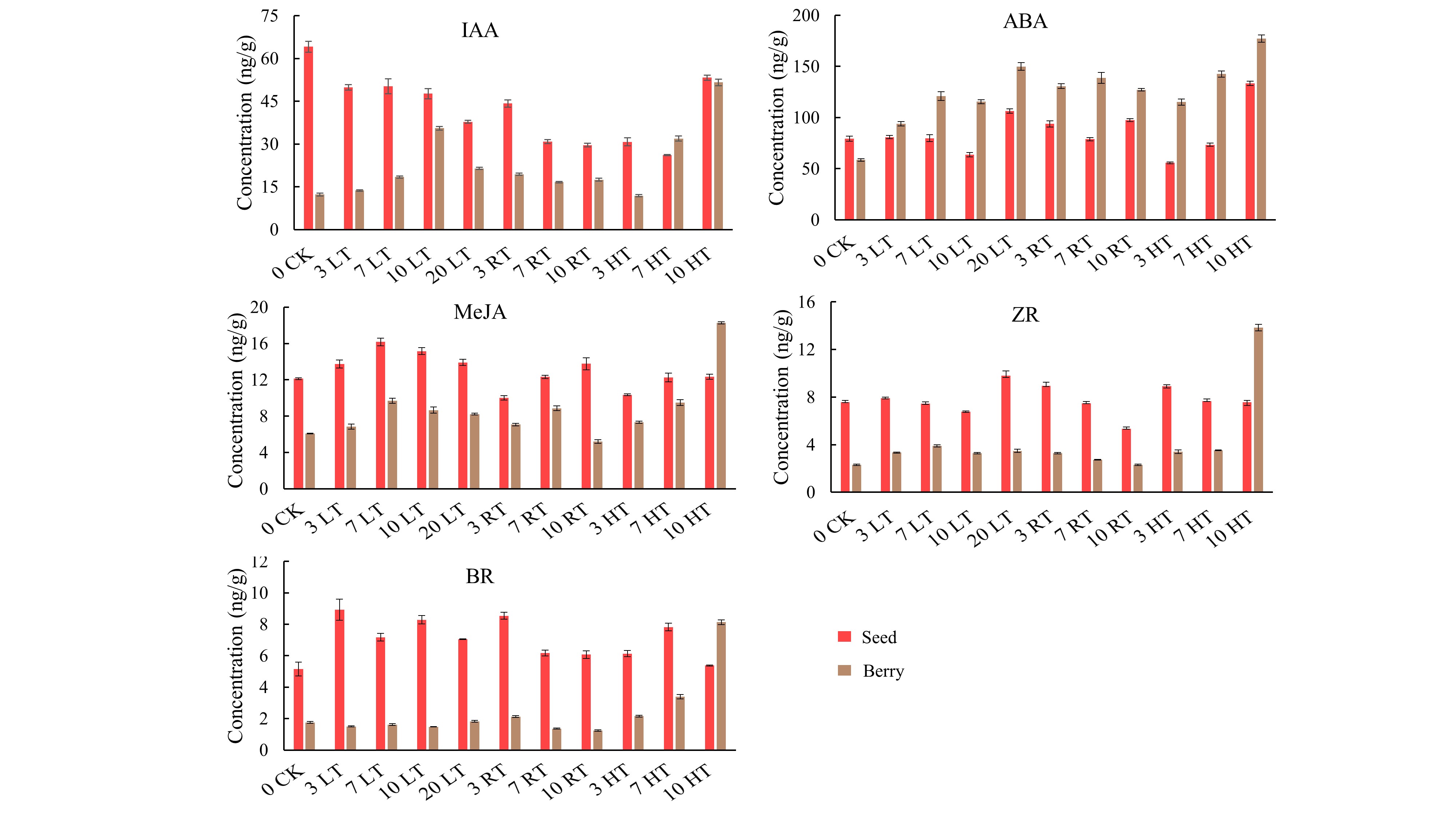


**Figure S3 Strawberry aroma components mass spectrum at 4 °C, 23 °C and 37 °C.** P1, P2 and P3 reprented proteins of sugar phosphate/phosphate translocator; 1-aminocyclopropane-1-carboxylate oxidase and aquaporin PIP2-2, respectively. LT, RT and HT represent the storage temperature of 4 °C, 23 °C and 37 °C, respectively. The red arrows represent a increase in aroma content after protein overexpression; the blue arrows represent new aroma components with a higher content after protein overexpression.


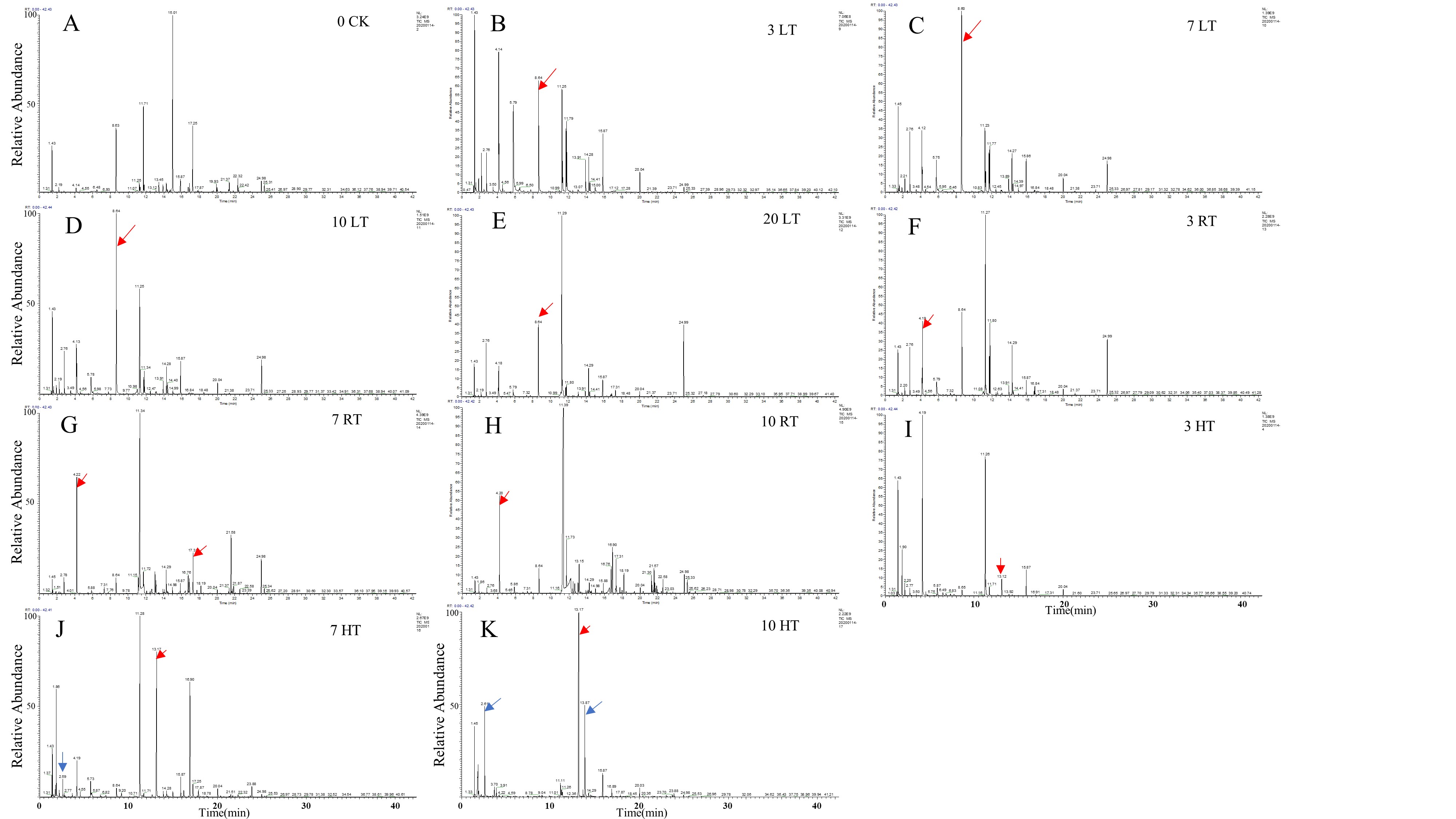


**Figure S4 Cell wall component content of strawberry at 4 °C, 23 °C and 37 °C.** (A) Hemicellulose content, (B) Cellulose content, (C) Soluble content and (D) Protopectin content. LT, RT and HT represent the storage temperature of 4 °C, 23 °C and 37 °C, respectively. Each value represents the mean of three replicates. Error bar stands for standard deviation (SD) and date are expressed as means ± SD.
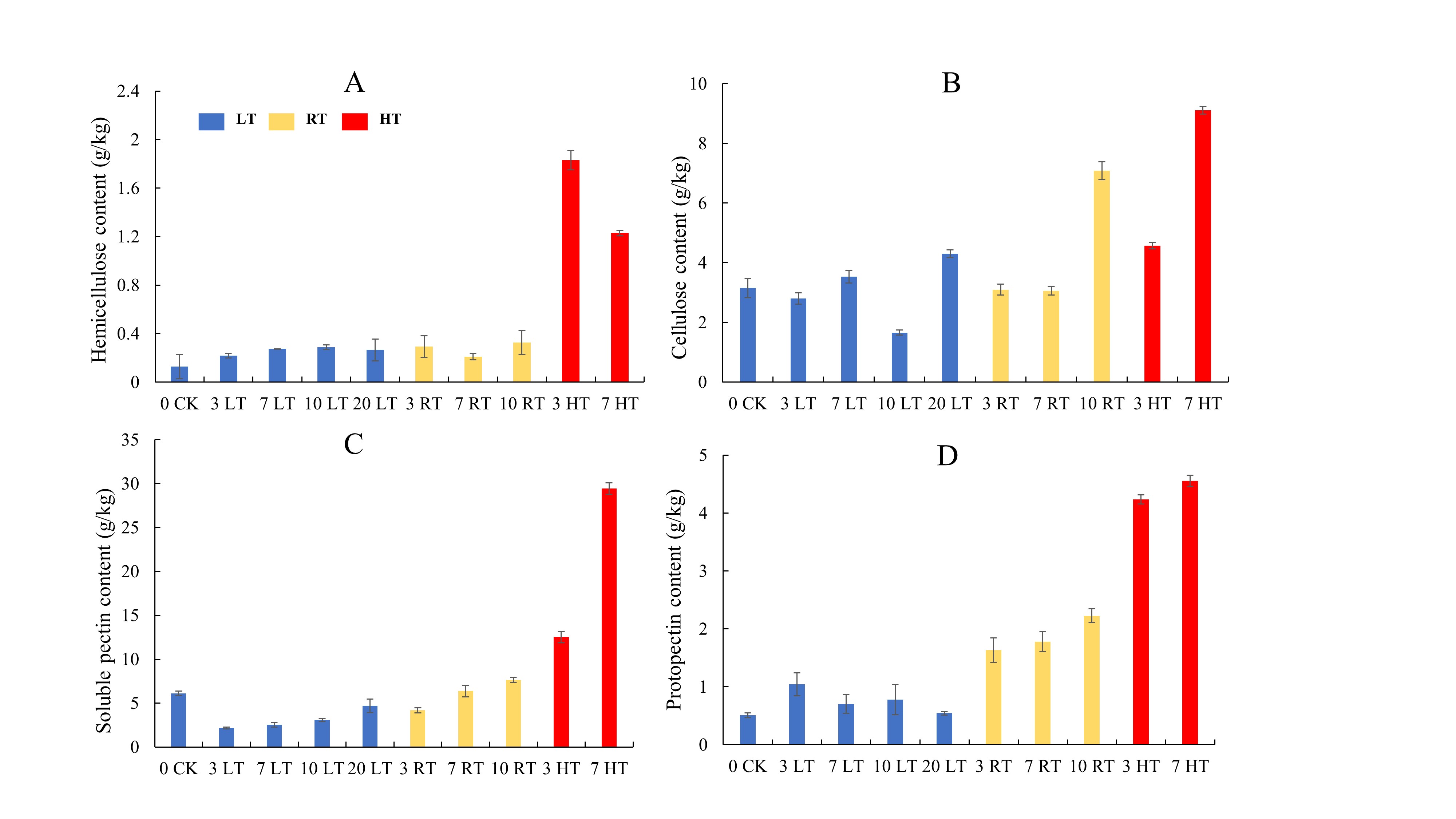


**Figure S5** Differentially expressed proteins (DEPs) in KEGG pathways **at 4 °C, 23 °C and 37 °C.** (A) Heat map of KEGG pathways, (B) HT vs RT, (C) LT vs RT, (D) LT vs HT. In Fig. A, the color blocks corresponding to the function description indicate the degree of enrichment. Red represents a strong degree of enrichment, and blue represents a weak degree of enrichment. In Fig. B, C and D, the abscissa is the name of the KEGG pathways, ordinate scale compared -log10 (p value), a schematic p value of 0.01 and 0.05 with red and green line, respectively. *P* > 0.05 represents significant difference and *P* > 0.01 represented a very significant difference. LT, RT and HT represent the storage temperature of 4 °C, 23 °C and 37 °C, respectively.


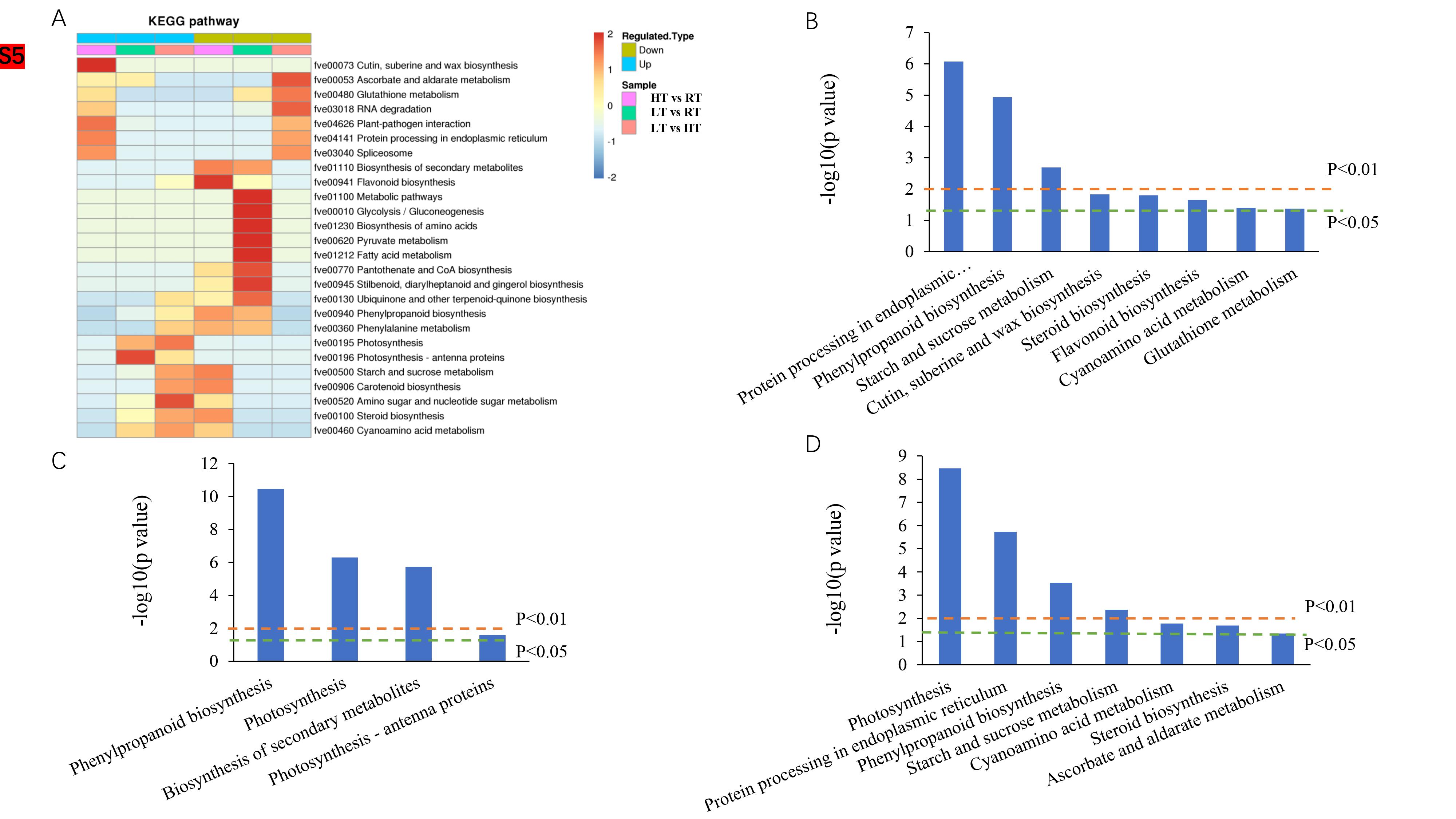


**Figure S6 Protein interaction network in different metabolism pathways at 4 °C, 23 °C and 37 °C.** Red circle dots represented significantly up-regulated proteins, and the darker the red, the higher the significance; blue represented significantly down-regulated proteins, and the darker the blue, the higher the significance; size of the circle represents the number of differential proteins and their interacting proteins. Boxes and ellipses indicate the position of heat shock protein. LT, RT and HT represent the storage temperature of 4 °C, 23 °C and 37 °C, respectively.


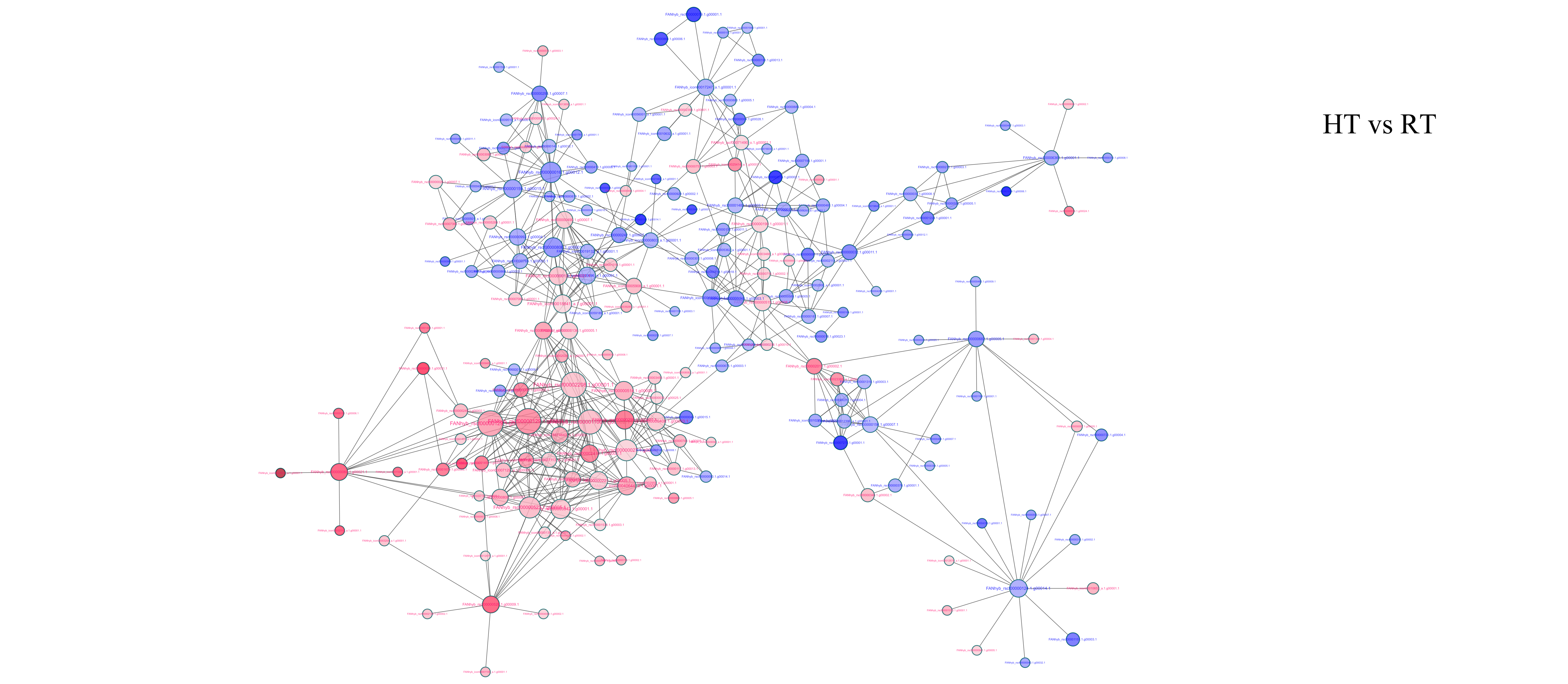

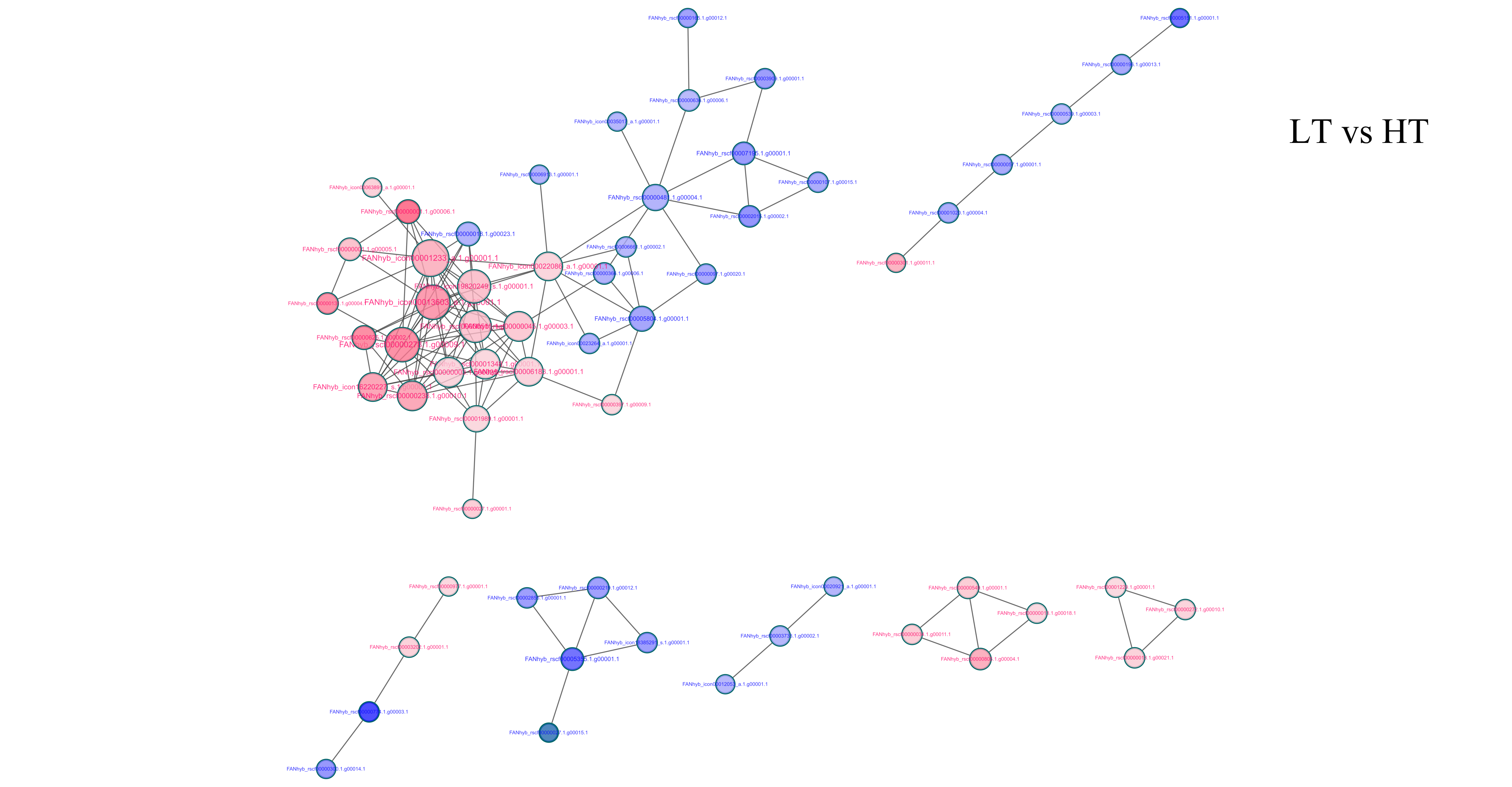


**Figure S7** **Relative gene expression level in strawberry at 4 °C, 23 °C and 37 °C.** (A) Expression level of anthocyanin biosynthesis related genes. (B) Expression level of cell wall related genes. ANS, anthocyanin synthase; CEL, cellulase; CHI, chalcone isomerase; CHS, chalcone synthase; DFR, dihydroflavonol-4-reductase; EXP, expansin. F3H, flavonoid-3-hydroxylase; GST, glutathione S-transferases; PAL, Phenylalanine/tyrosine ammonia-lyase; PE, pectinesterase; PG, polygalacturonase; PL, pectate lyases; UFGT, UDP-glucose flavonoid 3-O-glucosyltransferase; β-GAL, beta-galactosidase. LT, RT and HT represent the storage temperature of 4 °C, 23 °C and 37 °C, respectively. Each value represents the mean of three replicates. Error bar stands for standard deviation (SD) and date are expressed as means ± SD.**
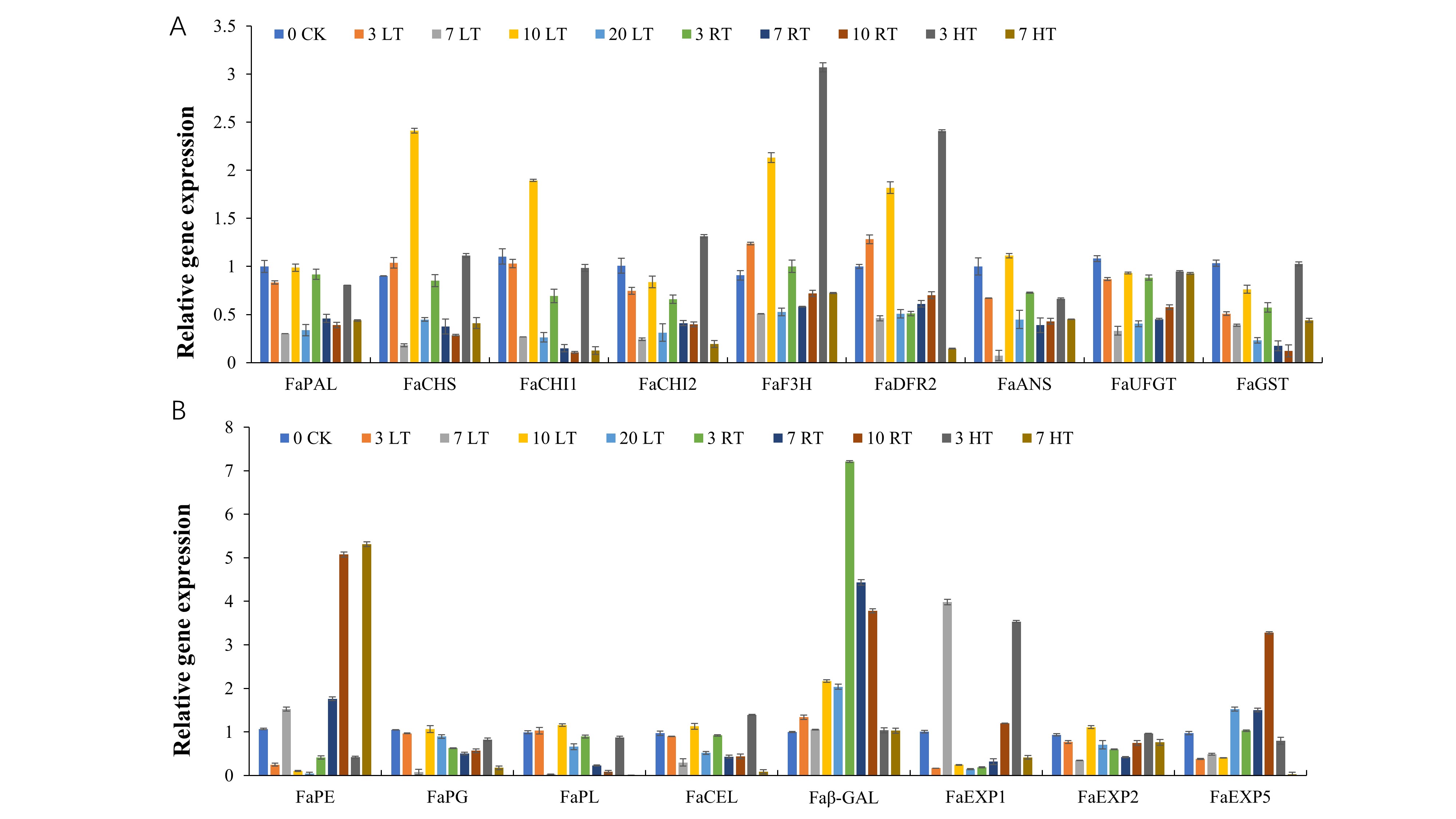
**


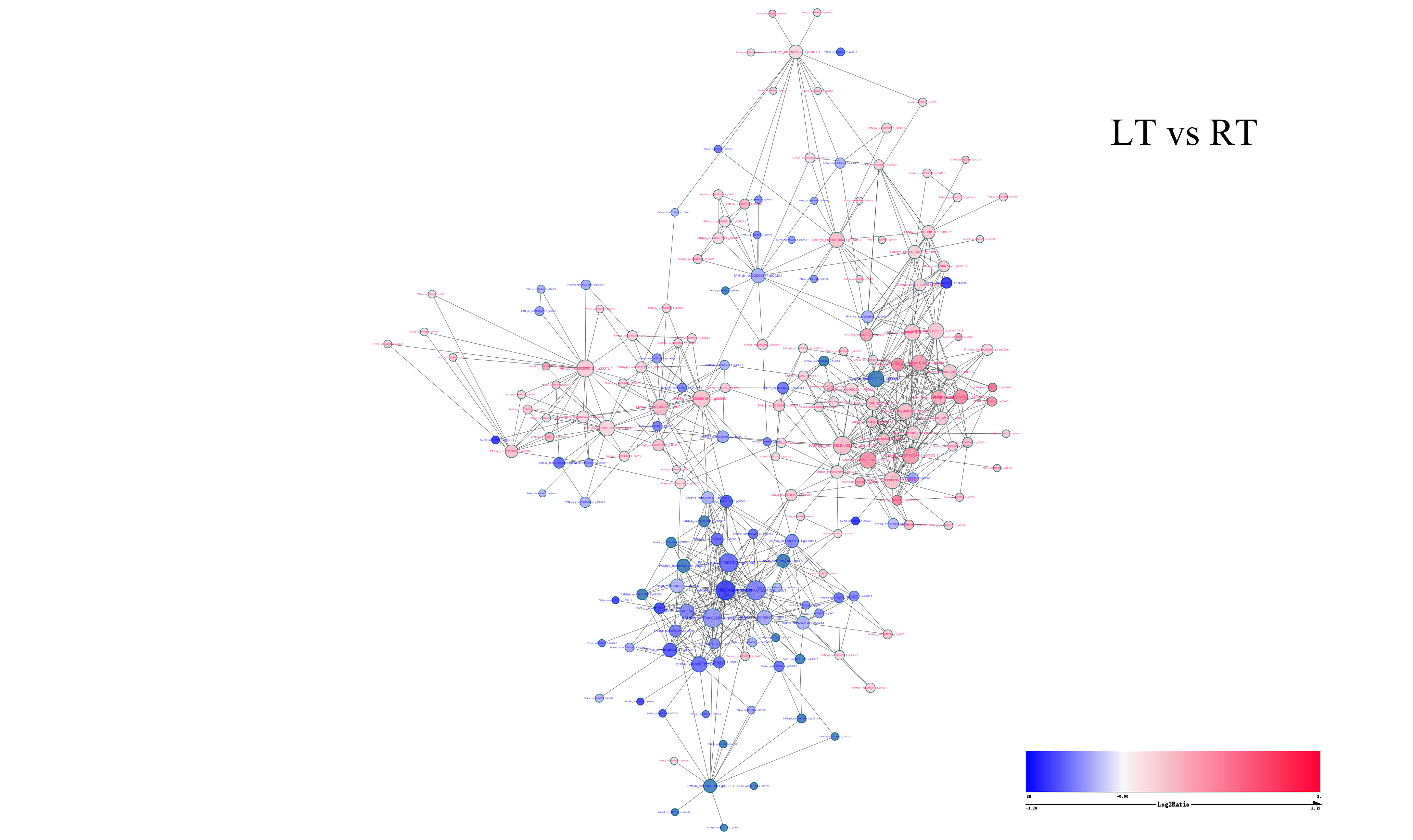


**Figure S8** **Strawberry aroma components mass spectrum after proteins overexpression at 4 °C, 23 °C and 37 °C.** P1, P2 and P3 reprented proteins of sugar phosphate/phosphate translocator; 1-aminocyclopropane-1-carboxylate oxidase and aquaporin PIP2-2, respectively. LT, RT and HT represent the storage temperature of 4 °C, 23 °C and 37 °C, respectively. The red arrows represent a increase in aroma content after protein overexpression.


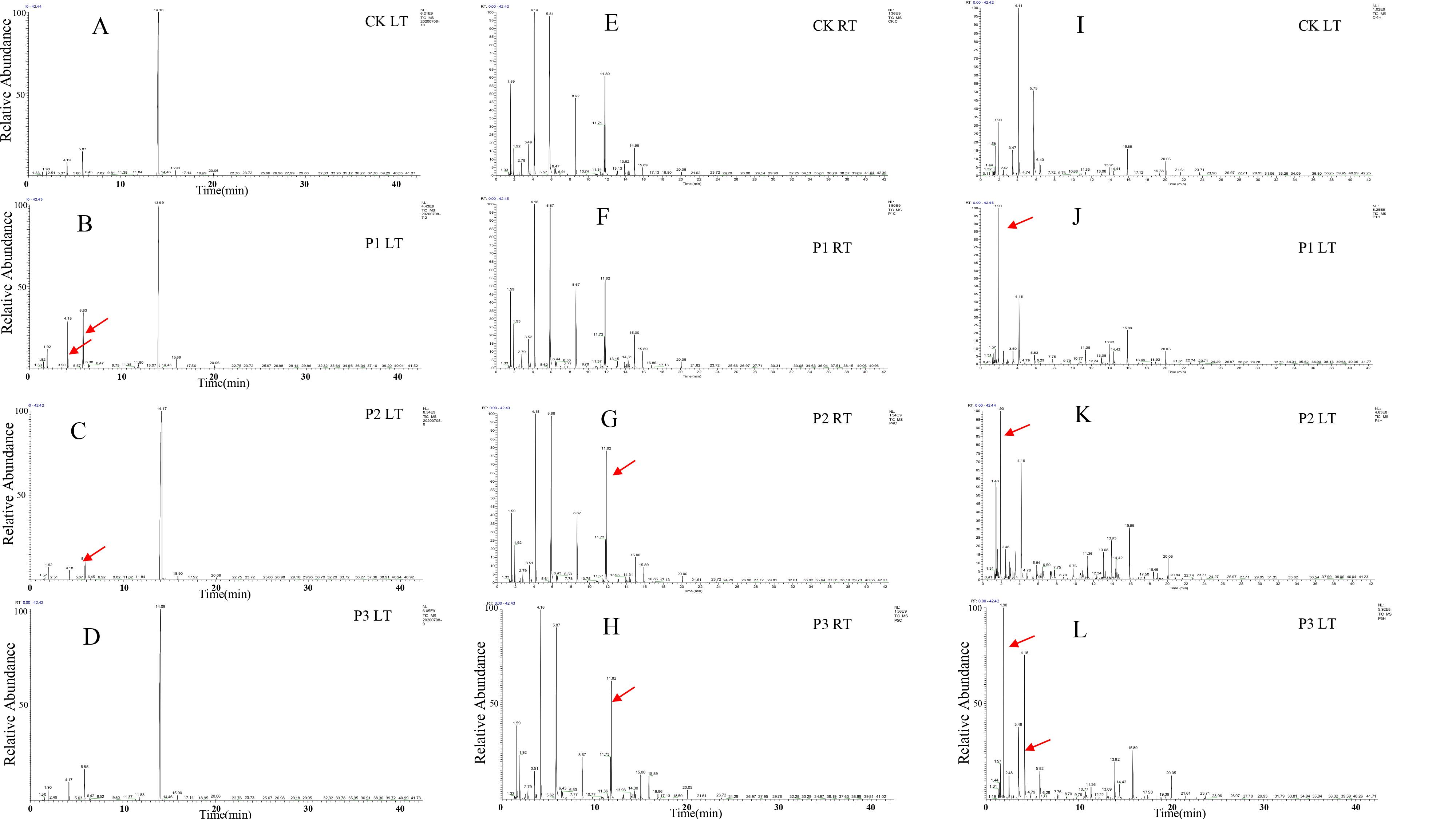


**Figure S9 Cell wall component content and antioxidant enzyme activity after proteins overexpression at 4 °C, 23 °C and 37 °C.** (A) Hemicellulose content, (B) Cellulose content, (C) Soluble content, (D) Protopectin content, (E) SOD activity and (F) POD activity. P1, P2 and P3 reprented proteins of sugar phosphate/phosphate translocator; 1-aminocyclopropane-1-carboxylate oxidase and aquaporin PIP2-2, respectively. LT, RT and HT represent the storage temperature of 4 °C, 23 °C and 37 °C, respectively. Each value represents the mean of three replicates. Error bar stands for standard deviation (SD) and date are expressed as means ± SD.
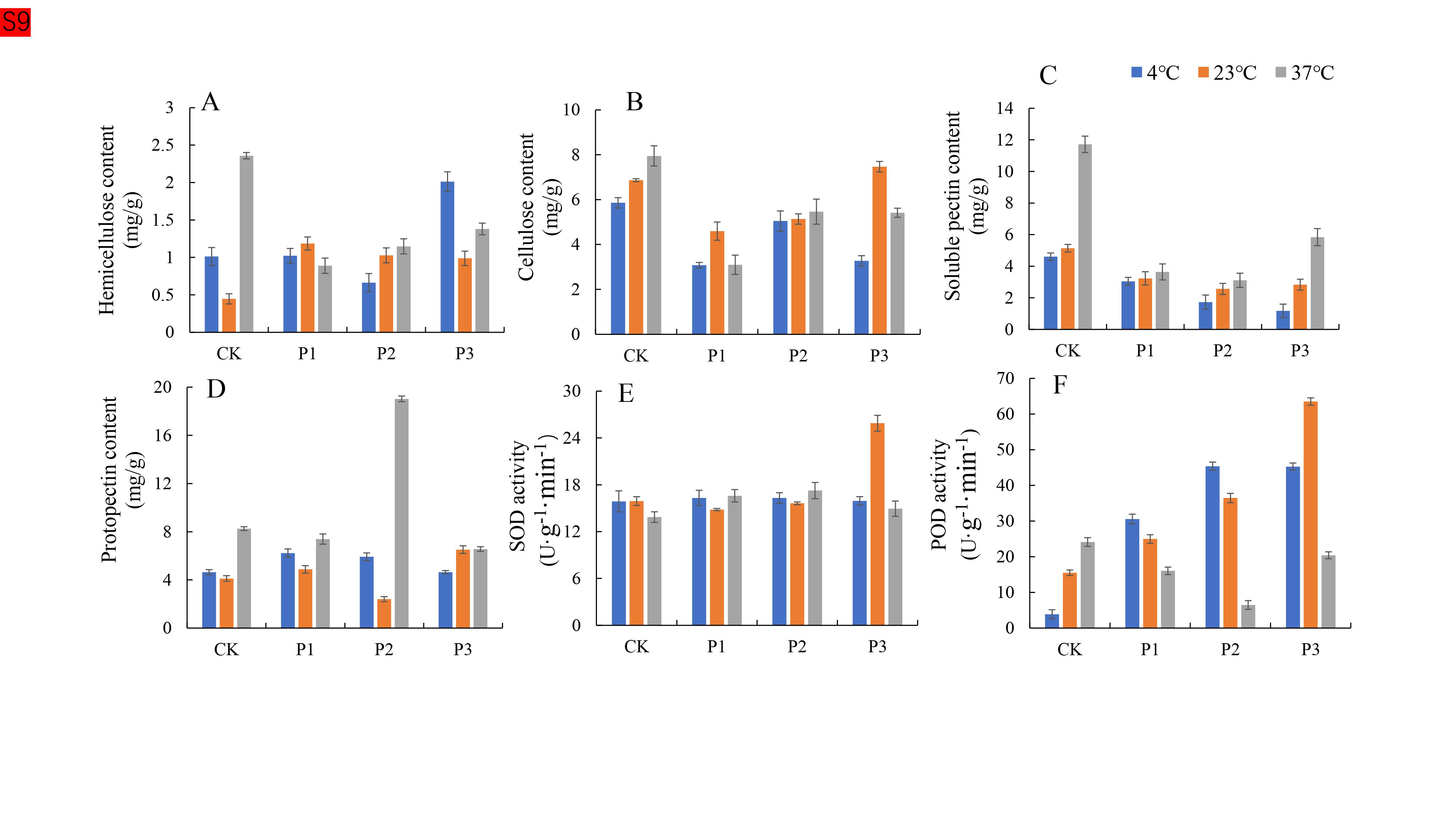


**Figure S10 Effect of proteins overexpression on relative gene expression level at 4 °C, 23 °C and 37 °C.** (A) Genes expression levels at 4 °C. (B) Genes expression levels at 23 °C. (C) Genes expression levels at 37 °C. P1, P2 and P3 reprented proteins of sugar phosphate/phosphate translocator; 1-aminocyclopropane-1-carboxylate oxidase and aquaporin PIP2-2, respectively. Error bar stands for standard deviation (SD). ANS, anthocyanin synthase; CEL, cellulase; CHI, chalcone isomerase; CHS, chalcone synthase; DFR, dihydroflavonol-4-reductase; EXP, expansin. F3H, flavonoid-3-hydroxylase; GST, glutathione S-transferases; PAL, Phenylalanine/tyrosine ammonia-lyase; PE, pectinesterase; PG, polygalacturonase; PL, pectate lyases; UFGT, UDP-glucose flavonoid 3-O-glucosyltransferase; β-GAL, beta-galactosidase. Each value represents the mean of three replicates. Error bar stands for standard deviation (SD) and date are expressed as means ± SD.LT, RT and HT represent the storage temperature of 4 °C, 23 °C and 37 °C, respectively.


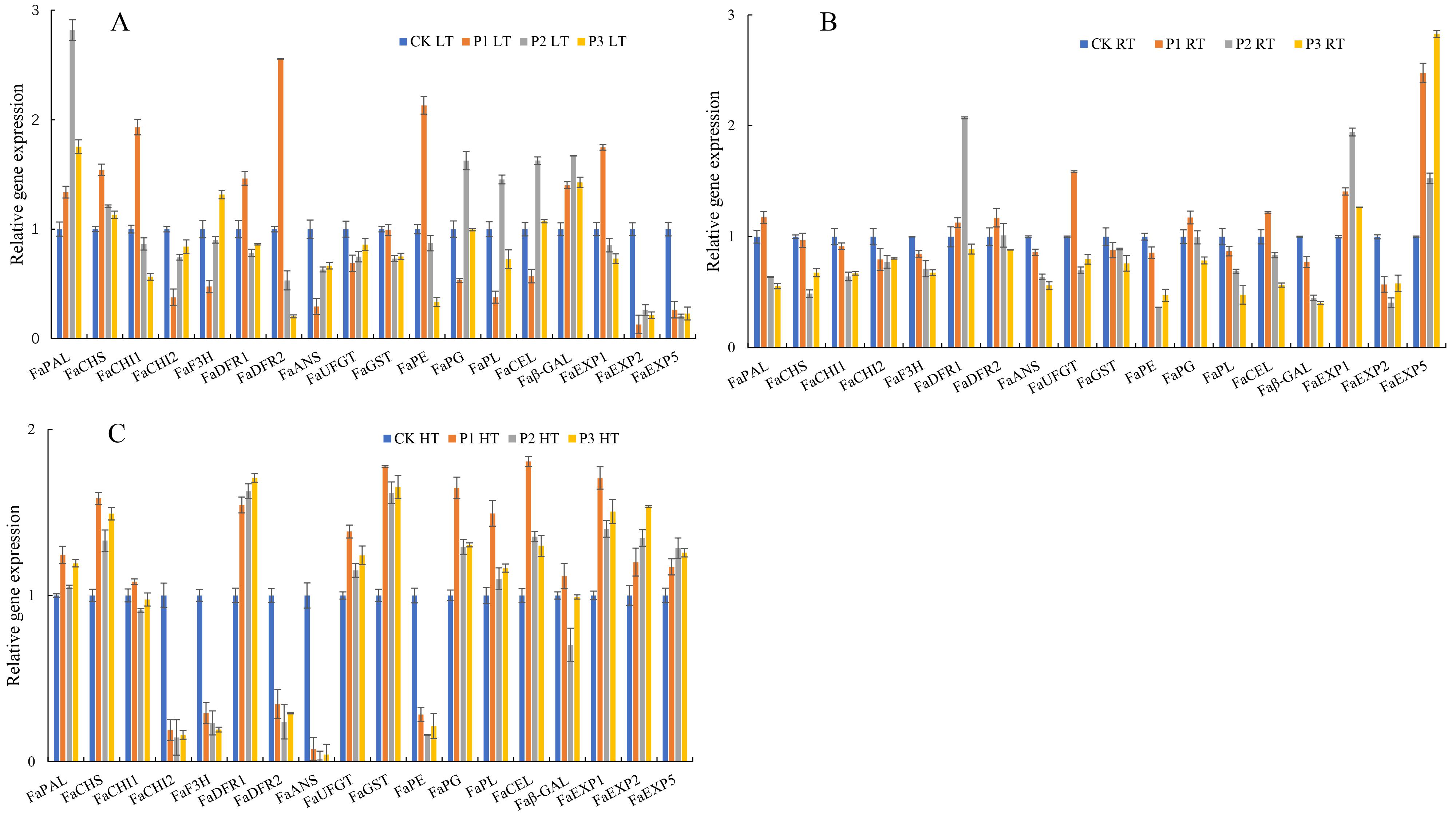

Supplement: Supplementary Figure 1 — Amino acid content in seed and berry of strawberry at 4, 23, and 37°C. [file Data_Sheet_1.docx]
